# Defining clusters of young autistic and typically developing children based on loudness-dependent auditory electrophysiological responses

**DOI:** 10.1186/s13229-020-00352-3

**Published:** 2020-06-15

**Authors:** Patrick Dwyer, Xiaodong Wang, Rosanna De Meo-Monteil, Fushing Hsieh, Clifford D. Saron, Susan M. Rivera

**Affiliations:** 1 Department of Psychology, UC Davis, One Shields Avenue, Davis, CA 95616 USA; 2 Center for Mind and Brain, UC Davis, 267 Cousteau Place, Davis, CA 95618 USA; 3 Department of Statistics, UC Davis, One Shields Avenue, Davis, CA 95616 USA; 4 MIND Institute, UC Davis, 2825 50th Street, Sacramento, CA 95817 USA

**Keywords:** Autism, Heterogeneity, Hierarchical clustering, Event-related potentials (ERPs), Subgroups, Sensory processing

## Abstract

**Background:**

Autistic individuals exhibit atypical patterns of sensory processing that are known to be related to quality of life, but which are also highly heterogeneous. Previous investigations of this heterogeneity have ordinarily used questionnaires and have rarely investigated sensory processing in typical development (TD) alongside autism spectrum development (ASD).

**Methods:**

The present study used hierarchical clustering in a large sample to identify subgroups of young autistic and typically developing children based on the normalized global field power (GFP) of their event-related potentials (ERPs) to auditory stimuli of four different loudness intensities (50, 60, 70, 80 dB SPL): that is, based on an index of the relative strengths of their neural responses across these loudness conditions.

**Results:**

Four clusters of participants were defined. Normalized GFP responses to sounds of different intensities differed strongly across clusters. There was considerable overlap in cluster assignments of autistic and typically developing participants, but autistic participants were more likely to display a pattern of relatively linear increases in response strength accompanied by a disproportionately strong response to 70 dB stimuli. Autistic participants displaying this pattern trended towards obtaining higher scores on assessments of cognitive abilities. There was also a trend for typically developing participants to disproportionately fall into a cluster characterized by disproportionately/nonlinearly strong 60 dB responses. Greater auditory distractibility was reported among autistic participants in a cluster characterized by disproportionately strong responses to the loudest (80 dB) sounds, and furthermore, relatively strong responses to loud sounds were correlated with auditory distractibility. This appears to provide evidence of coinciding behavioral and neural sensory atypicalities.

**Limitations:**

Replication may be needed to verify exploratory results. This analysis does not address variability related to classical ERP latencies and topographies. The sensory questionnaire employed was not specifically designed for use in autism. Hearing acuity was not measured. Variability in sensory responses unrelated to loudness is not addressed, leaving room for additional research.

**Conclusions:**

Taken together, these data demonstrate the broader benefits of using electrophysiology to explore individual differences. They illuminate different neural response patterns and suggest relationships between sensory neural responses and sensory behaviors, cognitive abilities, and autism diagnostic status.

## Background

Differential sensory processing in autism spectrum development (ASD)^1^ has historically been under-recognized and under-studied. Sensory processing was not listed as a clinical symptom of autism until the release of *DSM-5* in 2013 [2]. However, abundant evidence has emerged to highlight the relevance of differential sensory processing in ASD (see [3, 4] for reviews). For example, there is evidence of hyperacusis in autism [5–8]. The impact of these sensory differences depends upon autistic people’s environments [9], almost always designed with typical development (TD) in mind. Thus, differential sensory processing in ASD is associated with participation in activities [10, 11], adaptive functioning [12–15], anxiety and other affective symptoms [16–19], and even quality of life [20].

### Heterogeneity in autistic sensory processing

Sensory processing in ASD is also extremely heterogeneous [21]. First-person accounts demonstrate this diversity within the auditory domain. One autistic individual describes hypersensitivity to sudden, uncontrollable loud sounds like balloons and alarms (p. 69) [22]. Another reports struggling to tolerate soft echoing sounds in large spaces, like the rustling of papers (p. 74) [23], while yet another complains of how the sustained noise of crowds in hallways “seemed to make everything around me ‘echo’” [24]. Thus, different individuals have different patterns of auditory hyper-sensitivity and sensory interests, as well as unique experiences in response to different sounds. Furthermore, autistic people report that their individual sensory experiences can vary based on the degree to which the perceiver can predict or control the sensory stimuli, as well as the perceiver’s levels of anxiety and stress [25, 26]. These illustrations make clear the need for research which takes the heterogeneity of sensory processing in ASD into account.

### Caregiver- and self-report questionnaires

In recent years, researchers have started to use caregiver-report questionnaires to explore this heterogeneity, including studies which have clustered data from caregiver-report measures to identify sensory subtypes in ASD (for a systematic review, see [27]). However, two limitations of these studies are that they do not yield information about the neural processes that underlie sensory experiences and that caregivers cannot know directly what their children experience (see also [22], pp. 80–83). Indeed, although self-reported and parent-reported auditory hypersensitivities are significantly correlated in autistic adolescents without intellectual disabilities [28], the relatively low magnitude of the correlation coefficient (.49) obtained between parent- and self-reports on the same measure suggests that there are substantial differences between reporters.

### Neurophysiology

At present, relatively few studies using EEG or magnetoencephalography to investigate sensory processing in ASD have investigated heterogeneity. No published neurophysiological studies have attempted to separate autistic individuals into subgroups based neural markers of sensory processing, although some studies have reported associations between neurophysiological responses and various other variables in ASD [29–34], including other measurements of sensory processing, such as questionnaires [35–39].

Of the different ERP paradigms used to study autistic sensory processing, the presentation of tones of differing loudness (as used by [40, 41]), may hold particular promise in the exploration of heterogeneity. This approach was recently used to explore heterogeneity through the identification of differences between autistic participants with and without disproportionate megalencephaly [42]. Reports that some autistic people experience sensitivities to relatively soft sounds, while others describe greater sensitivity to louder sounds, suggest that exploring brain responses to stimuli of different loudness could reveal important information about individual differences in sensory processing in ASD.

### Heterogeneity in both ASD and typical sensory processing

It is difficult to fully interpret and contextualize the heterogeneity of sensory processing in ASD without also having some understanding of the heterogeneity of sensory processing in TD—and, in some ways, we know less about sensory heterogeneity in TD than ASD. Little and colleagues [43] explored sensory subtypes using parent reports in both autistic and typically developing participants and using self-reports; Elwin et al. [44] also characterized sensory subtypes in the general population and in ASD. These existing studies seem to suggest the existence of a single relatively “typical” subgroup dominated by typically developing participants (though including some autistic individuals), as well as an unclear number of additional subtypes that include more autistic participants. However, this literature remains limited.

### Present study

The present study is part of the Autism Phenome Project (APP) at the UC Davis MIND Institute, a large interdisciplinary study that aims to define subtypes of ASD based on behavioral, biochemical, and neurobiological indices. As part of the APP, electrophysiological responses to auditory stimuli of different intensities were collected from large numbers of participants at the first time-point after study entry. The present study aims to begin exploring neural heterogeneity in autistic and typical sensory processing by using these responses to define clusters of children with similar patterns of intensity-dependent auditory processing. In this study, the autistic and typically developing groups are combined and clustered in the same analysis so that sensory responses in each diagnostic group can be situated and understood in relation to one another. Furthermore, clusters are statistically compared to determine whether levels of different measured variables (such as cognitive ability and caregiver-reported sensory behavior) differ across clusters.

While the present study is exploratory, we made several predictions. First, as the clustering technique explicitly aims to group participants based on relative electrophysiological responses to stimuli of differing intensity, we expect that clusters will differ in their profiles of responses to stimuli of differing intensity. Second, owing to the existence of autistic individuals with sensory processing scores in the TD range in prior work (e.g., [43]), we anticipate that there will be substantial overlap between autistic and typically developing participants in the clusters defined in the analysis. Third, despite the substantial overlap predicted by the second hypothesis, we expect that there will be some separation of autistic and typically developing participants across clusters. Although research suggests that inter-individual variability in intensity-dependent cortical electrophysiological responses is considerable even in TD (e.g., [45, 46]), averaged response patterns in TD show monotonic increases of neural response strength with stimulus intensity [47]. In line with the patterns of subgroups found in questionnaire-based investigations of sensory heterogeneity in ASD and TD [43, 44], it would seem reasonable to anticipate that autistic participants might be more likely to display responses diverging from this monotopic grand-average pattern: that is, autistic participants might fall in clusters with unexpectedly strong responses to weak stimuli or displaying unexpectedly large increases in response strength to loud stimuli. This would also be consistent with some autistic people’s descriptions of particular subjective sensitivity towards the sounds of different loudness. Fourth, we expect that parents of participants in clusters with unexpectedly strong or weak responses to stimuli of particular intensities will report more atypical auditory sensory behaviors. Conversely, we predict that participants in clusters characterized by a relationship between stimulus and neural response strength similar to the overall grand-average will be rated as showing more typical sensory behaviors.

A considerable number of measures were collected as part of the APP. Given that anxiety [16–19], adaptive functioning [12–15], and cognitive ability [48, 49] have all been associated with autistic sensory processing in previous studies, and in light of the extensive literature documenting relationships between chronological age and electrophysiological responses in TD, these variables were also explored in this study.

## Methods

### Participants

As part of the APP, attempts were made to collect electrophysiological data from a total of 243 autistic and 96 typically developing children. All autistic participants met DSM-IV/Collaborative Programs for Excellence in Autism criteria for a Pervasive Developmental Disorder and passed cut-off scores on the ADOS-G [50] and, for either Social or Communication subscales, on the ADI-R [51]. Further details regarding the APP and participant recruitment can be found in previous publications (e.g., [52, 53]). Some participants were excluded from the present study due to noisy data, an insufficient number of acceptable-quality trials (< 400), an excessive number of excluded or poor-quality channels (> 6–7), or the presence of neuroanatomical abnormalities revealed by magnetic resonance imaging collected in the APP. One participant entered the study in the typically developing group but was diagnosed with autism at a later APP time-point; this participant’s data are also excluded. The final sample of children with usable electrophysiological data included a total of 81 typically developing participants (52 male) and 132 autistic participants (111 male) (Table 1). Families received a gift card in return for their participation in the study.

**Table 1:** Characteristics of typically developing and autistic participants with usable electrophysiological data

	TD		ASD	
	Mean (SD)	Range	Mean (SD)	Range
Chronological age (months)	37.09 (6.46)	25.80–56.33	38.54 (6.02)	25.50–54.87
MSEL Developmental Quotient (DQ)	106.36 (11.57)	79.89–128.62	64.83 (20.49)	30.39–132.45
MSEL Verbal DQ (VDQ)	107.97 (12.70)	81.26–149.47	58.40 (25.55)	19.31–127.98
MSEL Non-Verbal DQ (NVDQ)	104.75 (13.86)	71.49–129.96	71.26 (18.41)	36.39–136.93
VABS Adaptive Behaviour Composite	111.22 (12.00)	82.00–135.00	75.35 (11.00)	53.00–104.00

### Measures

#### Mullen Scales of Early Learning

Cognitive and communication assessments, as well as a number of caregiver-report questionnaires, were collected from participants as part of the APP. These measures included the Mullen Scales of Early Learning (MSEL) [54], a standardized measure of cognitive and motor functioning for children under the age of 68 months. Four MSEL subscales were administered: visual reception, fine motor, expressive language, and receptive language. A ratio developmental quotient (DQ) was calculated (as (mental age/chronological age)×100) for full-scale performance, as well as for verbal (VDQ) and nonverbal (NVDQ) performance. MSEL data were available from all 132 autistic participants with usable electrophysiological data, and for 80 of the 81 typically developing participants.

#### Vineland Adaptive Behavior Scales

These measures also included the parent-report form of the Vineland Adaptive Behavior Scales, Second Edition (VABS-II) [55], a rating scale designed for the assessment of adaptive functioning in populations with developmental disabilities. A standardized composite adaptive behavior score can be calculated. Complete VABS data were available from 117 ASD (96 male) and 69 TD participants (43 male).

#### Short Sensory Profile

The Short Sensory Profile (SSP) [56] was also collected. The SSP is a 38-item caregiver-report questionnaire that has been used in a number of studies to investigate and characterize autistic sensory processing (e.g., [18, 19, 48, 57, 58]). Higher scores reflect relatively typical sensory behaviors, whereas lower scores are indicative of atypicality. Complete SSP data were available from 99 of the 132 autistic participants, while partial data were available from 108 autistic participants (90 male). Complete SSP data were only available from 65 of the 81 typically developing participants, while some SSP subscales were available from a total of 66 (43 male). In addition to the original seven SSP subscales developed based on a typically-developing sample [56], two studies have explored SSP factors in samples of autistic children [59, 60]. In the present study, SSP items related to auditory sensory processing were of particular interest. Therefore, the nine-factor solution developed by Williams et al. [60], which specifically distinguishes behaviors reflecting auditory sensory sensitivity from those reflecting visual sensitivity, was selected. Given their relevance to the auditory domain, Williams et al.’s subscales for noise distress, auditory distractibility, and hyporesponsiveness to speech were examined in this study, along with the total score on all 38 SSP items.

#### Childhood Behavior Checklist

The preschool-age form of the Childhood Behavior Checklist (CBCL) was also collected [61]. This 100-item caregiver-report questionnaire aims to assess problematic internalizing and externalizing behaviors in young children. Of particular interest in the present study is the DSM-oriented anxiety problems subscale, given the previous reports of relationships between autistic sensory processing and anxiety. This subscale yields both a raw score and a normed T-score. CBCL DSM-oriented anxiety T-scores were available from 126 autistic (106 male) and 75 typically-developing (48 male) participants.

### Electroencephalography (EEG) task

The experimental setup and data collection have been previously described in [42]. Briefly, while seated on a caregiver’s lap in a dimly lit room, participants passively listened to 50 ms (including 5 ms rise and decay time) complex tones (sine waves of equal amplitude overlaid at the following 7 frequencies (musical notes): 249 Hz (B3); 616 Hz (D5), 788 Hz (G5), 1042 Hz (C6), 1410 Hz (F6), 1952 Hz (B6), and 2749 Hz (F7)) randomly presented at different intensities (50 dB, 60 dB, 70 dB, and 80 dB SPL) with a 1–2 s random ISI. The tones were presented via Sony MDR-222KD child-size headphones calibrated with a B&K artificial ear (model 4153) and sound meter (model 2229). Approximately ~1100–1200 trials were collected from each participant. Throughout the EEG recording, children watched a quiet video of their or their caregiver’s choice. Breaks were included as required, including briefly pausing the delivery of stimuli to suit child comfort.

### EEG data acquisition and processing

EEG data were collected with a 61-channel EASYCAP system [62] using a Compumedics Neuroscan Synamp II amplifier. Data were sampled at a rate of 1000 Hz with Cz as a reference. Data were then average-referenced and band-pass filtered with a low cutoff of 0.4 Hz offline (12 dB/octave roll-off). Epochs (spanning −200 ms to 900 ms, including 300 ms necessary for subsequent lagged correlations within an independent component analysis step) were screened and extreme amplitudes removed using the artifact scan tool of BESA 5.2 [63]. On average, in the ASD group, 23% of trials were removed in this process, compared to 19% in the TD group (see also Table 2). Given the study’s goal of exploring heterogeneity and individual differences in electrophysiological data, we sought to maximize the ERP signal-to-noise ratio by removing putatively non-neural signal sources from the data. To accomplish this, the remaining epochs were submitted to Second-Order Blind source Identification (SOBI; see [64, 65]). We used a semi-automatic artifact removal tool (SMART, [66]) to identify signal sources from SOBI that were interpreted, on the basis of signal source topography, spectra, autocorrelation and time series, to be of non-neural origin (such as electromyography/EMG, electrooculography/EOG, and blinks). Additional details regarding artifact removal using SOBI and SMART are discussed in [67]. Artifact-free trials were then reconstructed from the putatively neural SOBI signal sources and separate averages for each of the four loudness conditions were computed for each subject. Data from excluded channels were then interpolated using a 3-dimensional spline [68]. Epochs (now spanning 100 ms pre-stimulus onset to 600 ms post-stimulus onset) were filtered (second-order Butterworth with −12 dB/octave roll/off; 0.1 Hz high-pass; 40 Hz low-pass; 60 Hz notch) and baseline-corrected using the pre-stimulus period with the Cartool software [69].

**Table 2:** Means and standard deviations of number of trials retained in final averages. Means appear first, followed by standard deviations in brackets

	50 dB	60 dB	70 dB	80 dB
ASD	220.43 (50.50)	211.54 (52.00)	224.76 (49.93)	216.32 (49.97)
TD	240.07 (53.51)	229.49 (54.05)	244.00 (54.35)	234.63 (53.07)

The global field power (GFP) was used as an index of the strength of the brain’s response for analysis of the electrophysiological data. The GFP is computed as the standard deviation of all electrode channels per sample, and because the overall spatial distribution and gradients of the electrical potential across the montage are independent of the reference electrode, the GFP is reference-independent. Thus, GFP can be regarded as an index of the strength of the brain’s response to the stimulus, with higher GFP values reflecting a stronger neural response overall, independent of the spatial distribution of the electrocortical response [70]. We deemed this study’s focus on GFP appropriate because it allows for an entirely data-driven analysis, based on an index of the total strength of the neural response, that is not dependent upon the a priori selection of ERP components and electrode sites of interest. This may be of particular value in samples with young and neurodivergent children, some of whom may have unusual or idiosyncratic neural responses.

**Fig. 1:**
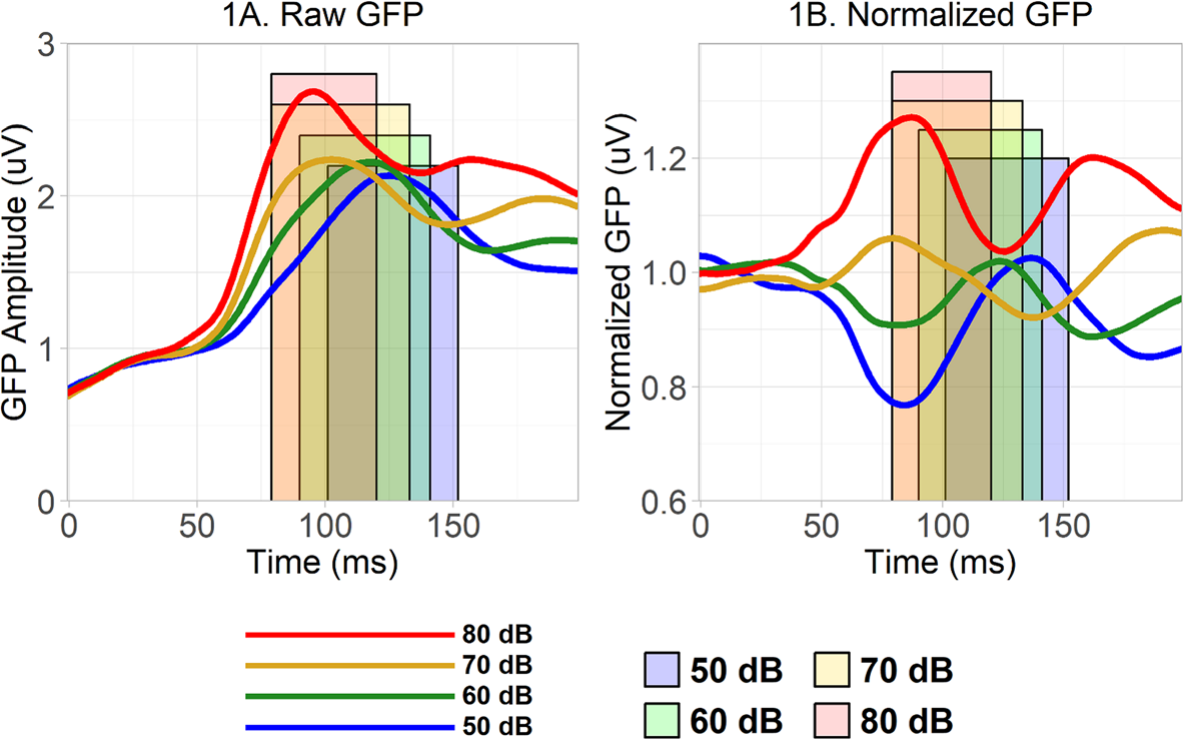
**a** Raw GFP from 0 to 200 ms post-stimulus onset averaged, separately in each loudness condition, across all participants in both diagnostic groups. The overlapping colored rectangles represent the different 85% fractional peak latency time windows from each of the loudness conditions. **b** Normalized GFP averaged across all participants in each loudness condition

To ensure that the clustering and correlation analyses focused explicitly on differences between loudness conditions (i.e., loudness-dependency) while ignoring the between participant absolute strength of the electrophysiological response across conditions, GFP for each participant in each condition was normalized. In the normalization process, each participant’s GFP value at each time-point in each condition was divided by that participant’s average GFP across all four conditions at the same time-point (see Fig. 1). Thus, the normalized GFP represents the strength of the neural response in a particular condition relative to the other conditions. It should be noted that this process should dramatically reduce the influence of non-neural factors that could contribute to individual differences in electrophysiological responses, such as the thickness of a participant’s skull [71].

### Hierarchical clustering analysis

The time windows used in the clustering analysis were defined by the criterion of ± 85% peak latency of the grand average of the raw GFP across groups. That is, in every loudness condition, separately, these were the time windows spanning the two time-points on either side of the raw GFP peak where raw GFP values were equal to 85% of the peak raw GFP. These windows, therefore, bracket the period of the strongest neural response. These windows corresponded to 101–152 ms for 50 dB; 90–141 ms for 60 dB; 79–133 ms for 70 dB; and 79–120 ms for 80 dB. Prior literature and visual inspection suggest that these periods correspond to the auditory P1, a large cortical auditory event-related response observed around ~100–150 ms in young children [72–74].

Next, the normalized GFP in the windows defined by 85% fractional peak latency was entered into a hierarchical clustering analysis. In this analysis, the normalized GFP values of each participant from each time-point in each loudness condition were clustered in R according to Ward’s agglomerative hierarchical method and visualized using heatmaps. Ward’s method aims to identify clusters based on distance in multivariate Euclidean space, by minimizing the variance within each cluster. That is, in Ward’s method, pairs of clusters are selected for merging based on the criterion that their merger should make the smallest possible increase in within-cluster variability. Initially, each cluster is a single participant, but clusters are then merged until all participants are grouped together. This generates a dendrogram which reflects the hierarchy of the clusters; participants appearing together in the lower branches are relatively less distant from (or more similar to) each other, in terms of the pattern of differences between loudness conditions, than participants that are widely separated in the dendrogram. Similar to K-means, Ward’s method utilizes object function- minimization of the within-cluster sum of squared error. However, Ward’s appears to be slightly more accurate than K-means in finding the number of clusters in datasets and uncovering relations among the clusters [75, 76]. Furthermore, in relation to the alternative hierarchical approach of single-linkage clustering, Ward’s method appears less susceptible to noise and less likely to produce elongated clusters [76, 77], although complete-linkage clustering has been shown to yield results more similar to Ward’s method [75].

**Fig. 2:**
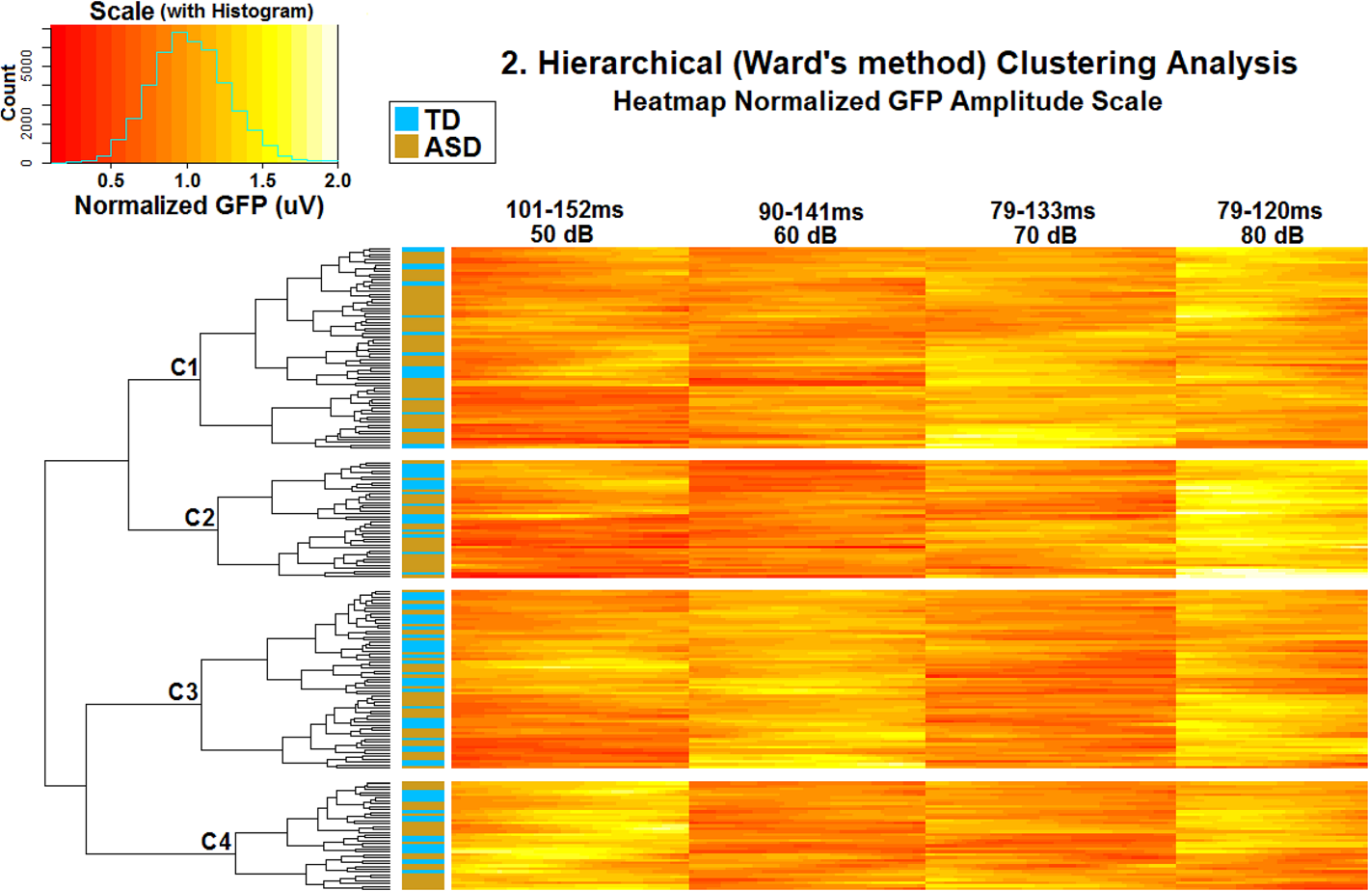
The hierarchical clustering analysis using Ward’s method. Each row is a participant, with autistic participants being marked as gold in the small column on the left, while typically developing participants are blue. The four main columns show normalized GFP in each loudness condition. Each column also depicts changes in normalized GFP over time, with earlier time-points being on the left of each column and later time-points on the right of each column. As shown in the scale in the upper left corner, smaller values, (reflecting a weaker normalized GFP in the loudness condition) are redder, while larger values (reflecting a stronger GFP) are yellower. A histogram showing the distribution of individual data points (individual data points represent a participant’s normalized GFP value in a given condition and at a given time-point) is superimposed over the scale. The dendrogram on the far left shows hierarchical clusters of similar participants. The horizontal lengths of the dendrogram branches represent the distance between clusters. The clusters selected for the purposes of this analysis are separated by blank space, and group numbering proceeds from top to bottom (i.e., C1 is at the top, C4 is at the bottom)

While clustering solutions are often selected on the basis of an optimization algorithm, Fushing and McAssey [78] demonstrate that the question of exactly how many clusters exist in a dataset is ill-posed. At least within (multi-)dimensional data clouds (rather than more naturally categorical data clouds with convex, well-separated clusters), different clusters can be said to exist at different hierarchical levels; it is hard to see how any single level necessarily yields the only valid, optimal solution. When clustering is used as a technique for exploring and describing dimensional data (as the authors believe loudness-dependent auditory responses should be regarded), a better question might be the question of what hierarchical level allows for the clearest description of the present dataset for the purposes of the present study. As such, the level of the dendrogram used for the determination of final cluster groups was manually selected based on the hierarchies formed in this analysis. A level of yielding four clusters was chosen (see Fig. 2). To further validate our clusters and offer at least a preliminary examination of their replicability albeit within the limits of the present dataset, subsamples of participants were repeatedly drawn and probabilities of reassignment alongside other participants from each original cluster were calculated (see Supplementary Methods and Results, Figures S1–S2).

#### Statistical comparisons of clusters.

A chi-square test of homogeneity was used to determine whether autistic participants were statistically more likely to fall into different clusters than typically developing participants, as well as vice versa. Omnibus effects were probed using adjusted standardized residuals (ASRs), which were obtained from each cell of the chi-square table and corrected for multiple comparisons using the Bonferroni-Holm procedure. With two diagnostic groups, comparisons in any row of the chi-square table perfectly mirrored one another, so the number of possible comparisons equaled the number of clusters.

To statistically compare the normalized GFP patterns found in different clusters, a parametric mixed ANOVA with loudness and cluster as factors was fitted. Interaction effects were explored further with follow-up one-way parametric ANOVAs separately in the 50 dB, 60 dB, 70 dB, and 80 dB conditions; results were corrected for multiple comparisons using the Bonferroni-Holm procedure. Afterwards, Welch’s *t*-tests, with a Bonferroni-Holm correction, were used to probe significant one-way ANOVA results. All results that reached significance prior to correction for multiple comparisons are reported with their corrected *p* values, but results that did not attain statistical significance before or after correction for multiple comparisons are not reported. Generalized eta-squared (${\eta}_G^2$) and Cohen’s *d* were used to estimate effect size.

Finally, to compare clusters and diagnostic groups on measured variables such as DQ and SSP scores, two-way between-groups ANOVA was used, unless assumptions of this parametric test were violated. If assumptions were violated, one-way Kruskal-Wallis tests were used, with no multiple comparison correction, to compare clusters separately in each diagnostic group. Wilcoxon-Mann-Whitney tests with Bonferroni-Holm corrections were used to determine the significance of post-hoc comparisons, which are reported using the effect size δ [79].

### Contiguity-based permutation correlation analyses

Due to the limitations of analyses involving comparison of levels of continuous variables across categorical subgroups defined on the basis of other continuous variables (see [80]), continuous associations were examined between normalized GFP and those variables which significantly differed across clusters in each diagnostic group, namely (as discussed below in the results): SSP total scores, SSP auditory distractibility scores, MSEL DQ, and MSEL NVDQ in ASD, as well as MSEL VDQ in TD. In each loudness condition, and at each time-point between 79 ms and 152 ms—that is, each time point falling into one of the windows used for the clustering analyses—Spearman’s rank-based correlation coefficient was used to examine the association between normalized GFP values and these variables. No multiple comparison correction was used to control for the number of different variables examined, so these tests are clearly exploratory. However, a contiguity-based permutation testing approach^2^ [81] was used to correct for the multiple comparisons entailed by separately examining effects at numerous consecutive time-points. First, all test statistics exceeding an initial two-tailed probability threshold of .05 were grouped into different temporally contiguous series of time points. Obtained statistics from these contiguous series were summed. To determine final statistical significance, summed statistics from the obtained contiguous series were compared against the null distribution of summed contiguous series statistics generated through 10,000 random permutations of the data. Essentially, this procedure discards any associations that are not stronger and/or more temporally sustained than would be expected from chance alone.

## Results

### Hierarchical clustering analysis

Grand-averaged raw and normalized GFP across both groups are depicted in Fig. 1, visual inspection of which shows that intensity systematically modulated neural responses, as well as supplementary Figures S3–S6, while the results of the hierarchical clustering analysis are displayed in Fig. 2. Groups are referred to as “C1” (referring to “Cluster 1”) through “C4”. Generally, analyses of cluster replicability (albeit within the present dataset) based on drawing and clustering repeated subsamples suggest the present clusters are fairly replicable, with participants being substantially more likely to be clustered alongside other participants from their original clusters than alongside those outside their original clusters (Supplementary Tables S2–S3), although it should be noted that one subgroup of participants within C1 showed some propensity to move back and forth between C1 and C2 on resampling (Supplementary Figures S1–S2).

### Diagnostic group membership

The distributions of autistic and typically-developing participants across the clusters differed significantly, *X*^2^ (3, *N* = 213) = 8.42, *p* = .04 (Table 3). C1 had significantly more autistic participants than expected based on the proportion of ASD participants in the study, ASR = 2.70, corrected *p* = .03. Furthermore, at a trend level, C3 had more typically developing participants than expected, ASR = 2.18, corrected *p* = .06.

**Table 3:** Counts and percentages of autistic and typically-developing participants, separately, in cluster groups

	C1	C2	C3	C4
ASD	53 (74.65%)	24 (58.54%)	32 (50.79%)	23 (60.53%)
TD	18 (25.35%)	17 (41.46%)	31 (49.21%)	15 (39.47%)

### Cluster electrophysiological patterns

**Fig. 3:**
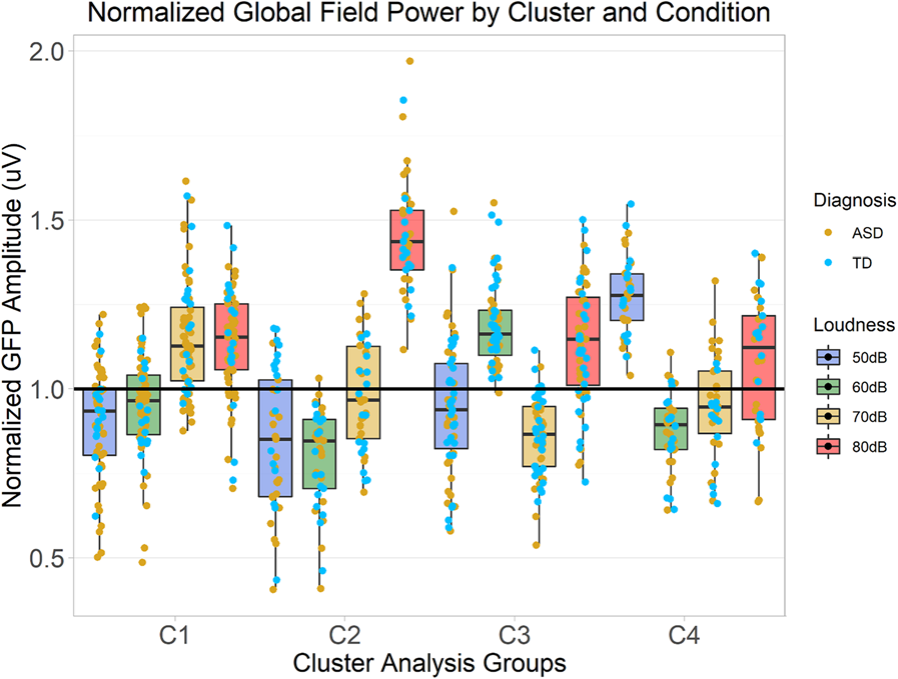
Normalized GFP averaged across clustering time windows in each loudness condition and cluster collapsed across both diagnostic groups. C1 contains 53 autistic and 18 typically developing participants, C2 contains 24 autistic and 17 typically developing participants, C3 contains 32 autistic and 31 typically developing participants, and C4 contains 23 autistic and 15 typically developing participants. Hinges (outer limits of boxes) correspond to first and third quartiles (25th and 75th percentiles) and whiskers extend either 1.5× the interquartile range outwards from the boxes, or the range of the data, whichever is smaller.

**Fig. 4:**
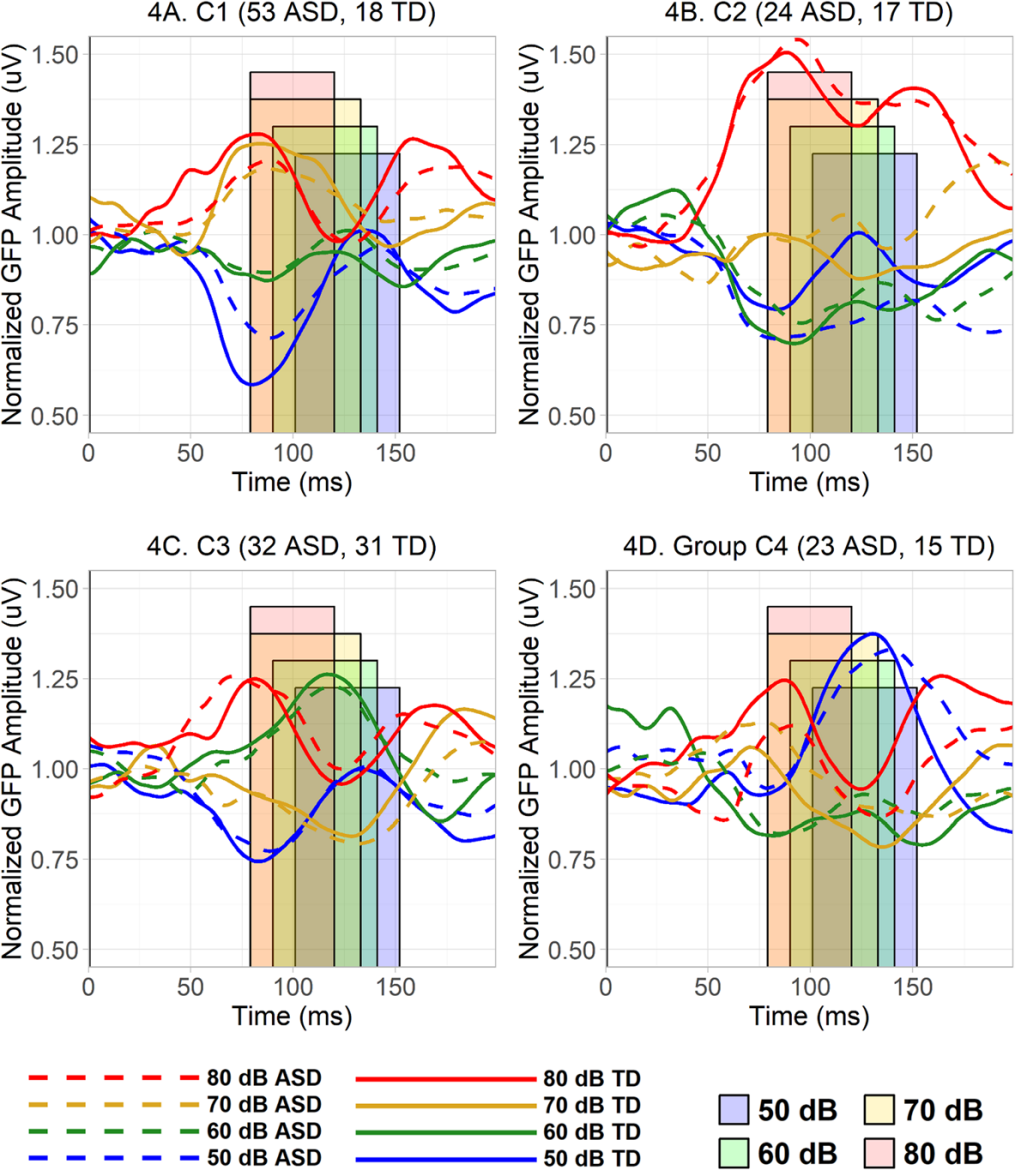
**a–d** Normalized GFP averaged, separately in each loudness condition and diagnostic group, across participants each group and cluster. The overlapping colored rectangles represent the different 85% fractional peak latency time windows from each of the different loudness conditions. Note that while normalized GFP patterns do differ across clusters, ASD and TD participants within each cluster appear similar. **a** Normalized GFP averaged, separately in each loudness condition and diagnostic group, across participants from C1. **b** Normalized GFP averaged across participants in each diagnostic group from C2. **c** Normalized GFP averaged across participants in each diagnostic group from C3. **d** Normalized GFP averaged across participants in each diagnostic group from C4

There was a significant between-subjects main effect of the cluster on normalized GFP, *F*(3, 209) = 3.86, *p* = .01, ${\eta}_G^2$ = .003, there was a significant within-subjects effect of loudness, *F*(3, 627) = 78.23, *p* < .0001, ${\eta}_G^2$ = .26, and there was a significant cluster by loudness interaction, *F*(9, 627) = 55.09, *p* < .0001, ${\eta}_G^2$ = .43 (Figs. 3 and 4). This robust interaction confirmed that the hierarchical clustering analysis succeeded in defining clusters that differed in the loudness-dependency of their electrophysiological responses to auditory stimuli.

In the 50 dB condition, normalized GFP significantly differed across the clusters, *F*(3, 209) = 49.09, corrected *p* < .0001, ${\eta}_G^2$ = .41. The brain’s electrophysiological response to 50 dB sounds was significantly stronger in C4 than in any other cluster group (compared to C1, Welch’s *t*(96.90) = 13.91, corrected *p* < .0001, *d* = 2.55; compared to C2, Welch’s *t*(63.42) = −11.36, corrected *p* < .0001, *d* = 2.50; and compared to C3, Welch’s *t*(98.99) = 10.58, corrected *p* < .0001, *d* = 1.93). There was also a trend for a stronger 50 dB response in C3 compared to C2, but this was not significant after applying the Holm-Bonferroni correction, Welch’s *t*(81.40) = 2.45, corrected *p* = .11, *d* = 0.50.

In the 60 dB condition, normalized GFP significantly differed across the clusters, *F*(3, 209) = 85.54, corrected *p* < .0001, ${\eta}_G^2$ = .55. The brain’s electrophysiological response to 60 dB sounds was significantly stronger in C3 than any other group (compared to C1, Welch’s *t*(131.85) = 10.04, corrected *p* < .0001, *d* = 1.72; compared to C2, Welch’s *t*(76.02) = 14.30, corrected *p* < .0001, *d* = 2.97; and compared to C4, Welch’s *t*(83.34) = 12.91, corrected *p* < .0001, *d* = 2.60). Furthermore, the brain’s response to 60 dB sounds was stronger in C1 than C2, Welch’s *t*(83.44) = 5.64, corrected *p* < .0001, *d* = 1.11, and it was stronger in C1 than C4, Welch’s *t*(92.12) = 3.15, corrected *p* = .02, *d* = 0.59. A trend for the 60 dB response to be stronger in C4 than C2 did not survive post-hoc correction, Welch’s *t*(75.05) = 2.77, corrected *p* = .06, *d* = 0.62.

In the 70 dB condition, normalized GFP significantly differed across the clusters, *F*(3, 209) = 45.57, corrected *p* < .0001, ${\eta}_G^2$ = .40. The brain’s electrophysiological response to 70 dB sounds was significantly stronger in C1 than any other group (compared to C2, Welch’s *t*(88.25) = 5.48, corrected *p* < .0001, *d* = 1.06; compared to C3, Welch’s *t*(124.08) = 11.90, corrected *p* < .0001, *d* = 2.01; and compared to C4, Welch’s *t*(82.60) = 6.62, corrected *p* < .0001, *d* = 1.29). Furthermore, the brain’s response to 70 dB sounds was significantly weaker in C3 than in C2, Welch’s *t*(67.23) = –4.20, corrected *p* = .0009, *d* = −0.90, and the 70 dB response was significantly weaker in C3 than C4, Welch’s *t*(62.34) = −2.91, corrected *p* = .05, *d* = −0.64.

In the 80 dB condition, normalized GFP significantly differed across the clusters, *F*(3, 209) = 42.00, corrected *p* < .0001, ${\eta}_G^2$ = .38. The brain’s electrophysiological response to 80 dB sounds was significantly stronger in C2 than any other group (compared to C1, Welch’s *t*(76.23) = 9.74, corrected *p* < .0001, *d* = 1.97; compared to C3, Welch’s *t*(87.61) = 9.13, corrected *p* < .0001, *d* = 1.82; and compared to C4, Welch’s *t*(74.44) = 9.13, corrected *p* < .0001, *d* = 2.06). These effects were robust to the removal of the three outlying participants in C2 (based on the criterion of 3× the median absolute deviation) visible in Fig. 3.

### Measures and demographics

#### Caregiver-reported sensory symptoms

**Fig. 5:**
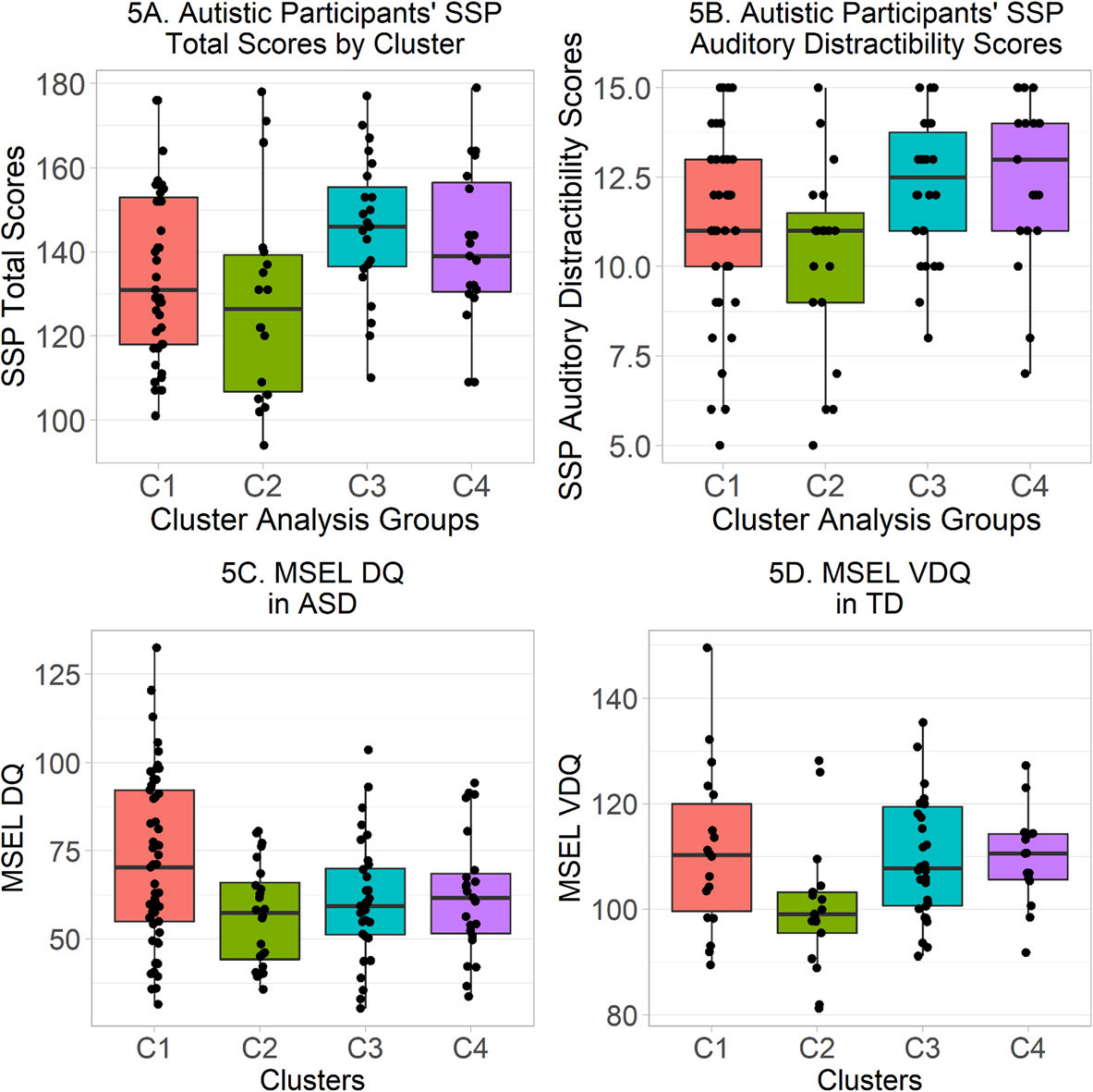
**a–d a** Total SSP scores in autistic participants from each cluster. Counting only those with complete SSP data, C1 contains 39 autistic participants, C2 contains 18 autistic participants, C3 contains 23 autistic participants, and C4 contains 19 autistic participants. Outer limits (hinges) of boxes correspond to first and third quartiles (25th and 75th percentiles) and whiskers extend either 1.5× the interquartile range outwards from the boxes, or the range of the data, whichever is smaller. **b** SSP Auditory Distractibility scores in autistic participants from each cluster. Counting only those with complete SSP Auditory Distractibility data, C1 contains 41 autistic participants, C2 contains 19 autistic participants, C3 contains 26 autistic participants, and C4 contains 19 autistic participants. **c** MSEL DQ in autistic participants from each cluster. **d** MSEL VDQ in typically developing participants from each cluster

Total scores on the SSP showed a non-normal distribution, Shapiro-Wilk *W* = .96, *p* = .0003, as did scores on the three SSP auditory factors defined by Williams et al. [60], Shapiro-Wilk *p* ≤ .0001. Furthermore, combining across clusters, autistic participants had significantly lower (i.e., more atypical) SSP total scores than typically developing participants, Wilcoxon-Mann-Whitney *W* = 746.5, *n*_ASD_ = 99, *n*_TD_ = 65, *p* < .0001, δ = −.88, and the same pattern was observed with all three auditory subscores, *p* ≤ .008. Therefore, one-way non-parametric Kruskal-Wallis tests were used to compare clusters on SSP scores and subscores separately in each diagnostic group. Among autistic participants, total SSP scores differed between clusters, *H*(3) = 8.67, *p* = .03 (Table 4, Fig. 5a). Wilcoxon-Mann-Whitney tests indicated that sensory processing trended towards being more atypical in C2 than C3, *W* = 108.5, *n*_C2_ = 18, *n*_C3_ = 23, corrected *p* = .06, δ = −.48. Furthermore, among autistic participants, scores on the SSP Auditory Distractibility factor significantly differed between clusters, *H*(3) = 10.31, *p* = .02 (Fig. 5b). Wilcoxon-Mann-Whitney tests indicated that more auditory distractibility was reported in C2 than C4, *W* = 90.5, *n*_C2_ = 19, *n*_C4_ = 19, Bonferroni-Holm corrected *p* = .05, δ = −.50; there was also a trend for more auditory distractibility in C2 than C3, *W* = 139.5, *n*_C2_ = 19, *n*_C3_ = 26, corrected *p* = .07, δ = −.44. Trends in the ASD sample for effects of the cluster on Noise Distress did not attain significance. In the typically developing sample, one-way Kruskal-Wallis tests revealed no significant differences between clusters on the SSP total score or any of the auditory subscores (Supplementary Table S1).

**Table 4:** SSP total scores and auditory subscores for autistic participants by cluster

	Cluster means (standard deviations)				Kruskal-Wallis test		Cliff’s δ^1^					
	C1	C2	C3	C4	*H*(3)	*p*	C1, C2	C1, C3	C1, C4	C2, C3	C2, C4	C3, C4
Auditory Distractibility	11.15 (2.56)	10.21 (2.70)	12.19 (1.92)	12.47 (2.37)	10.31	.02	.21	−.23	−.31	−.44	−.50*	−.13
Hyporesponsiveness to Speech	4.81 (1.52)	4.38 (2.11)	4.65 (1.38)	5.21 (1.65)	3.89	.27	.22	.05	−.10	−.19	−.35	−.15
Noise Distress	7.19 (2.33)	6.24 (2.61)	8.08 (1.79)	7.68 (1.92)	7.21	.07	.22	−.21	−.10	−.43	−.34	.13
Total SSP Score	135.08 (20.08)	128.50 (24.51)	145.43 (16.69)	141.42 (18.75)	8.67	.03	.19	−.30	−.21	−.48	−.37	.15

^1^Values of δ are indicated with * if corresponding the Wilcoxon-Mann-Whitney *p* value is < .05 after Bonferroni-Holm correction for six comparisons

#### Chronological age

There was no main effect of cluster group, *F*(3, 204) = 1.02, *p* = .38, ${\eta}_G^2$ = .01, or diagnostic group, *F*(1, 204) = 2.19, *p* = .14, ${\eta}_G^2$ = .01 on chronological age, nor was there an interaction of cluster and diagnostic groups, *F*(3, 204) = 0.90, *p* = .44, ${\eta}_G^2$ = .01.

#### Cognitive ability

MSEL DQ had a non-normal distribution in ASD, Shapiro-Wilk *W* = .96, *p* = .0008. Furthermore, autistic participants had significantly lower DQ than typically developing participants (Table 1). Therefore, one-way non-parametric Kruskal-Wallis tests were used to compare clusters on DQ, VDQ, and NVDQ scores separately in each diagnostic group. Among autistic participants, DQ scores differed between clusters, *H*(3) = 8.28, *p* = .04 (Table 5, Fig. 5c). Similar effects were found in NVDQ scores, and a similar trend was seen in VDQ scores, although in each case post-hoc comparisons failed to locate significant differences after strict corrections for multiple comparisons were applied. Among typically developing participants, VDQ scores significantly differed between clusters (Table 6, Fig. 5d), *H*(3) = 9.08, *p* = .03; after correction, scores in C2 were lower than C4, *W* = 56.0, *n*_C2_ = 17, *n*_C3_ = 15, corrected *p* = .04, δ = –.56. No effects of full-scale DQ and NVDQ were observed in TD.

**Table 5:** MSEL DQ, NVDQ, and VDQ scores by for autistic participants by cluster

	Cluster means (Standard deviations)				Kruskal-Wallis test		Cliff’s δ^1^					
	C1	C2	C3	C4	*H*(3)	*p*	C1, C2	C1, C3	C1, C4	C2, C3	C2, C4	C3, C4
MSEL DQ	71.89 (23.96)	56.73 (14.31)	60.88 (16.92)	62.54 (17.50)	8.28	.04	.37	.25	.22	−.11	−.19	−.05
MSEL NVDQ	77.38 (21.21)	64.83 (12.81)	68.16 (17.16)	68.19 (14.43)	9.00	.03	.36	.28	.27	−.10	−.11	−.11
MSEL VDQ	66.40 (29.09)	48.63 (18.92)	53.59 (22.05)	56.87 (23.11)	7.64	.05	.35	.25	.19	−.13	−.19	−.08

^1^Values of δ are indicated with * if corresponding the Wilcoxon-Mann-Whitney *p* value is < .05 after Bonferroni-Holm correction for six comparisons

**Table 6:** MSEL DQ, NVDQ, and VDQ scores by for typically developing participants by cluster

	Cluster means (Standard deviations)				Kruskal-Wallis test		Cliff’s δ^1^					
	C1	C2	C3	C4	*H*(3)	*p*	C1, C2	C1, C3	C1, C4	C2, C3	C2, C4	C3, C4
MSEL DQ	106.41 (11.80)	100.30 (11.75)	108.63 (10.75)	108.63 (11.33)	5.62	.13	.24	−.13	−.13	−.39	−.41	−.01
MSEL NVDQ	101.73 (13.13)	100.22 (13.29)	107.65 (13.08)	107.71 (15.99)	4.72	.19	.08	−.23	−.25	−.33	−.30	−.03
MSEL VDQ	111.09 (15.53)	100.38 (12.55)	109.62 (11.31)	109.55 (9.05)	9.08	.03	.45	.02	−.01	−.45	−.56*	−.02

^1^Values of δ are indicated with * if corresponding the Wilcoxon-Mann-Whitney *p* value is < .05 after Bonferroni-Holm correction for six comparisons

#### Adaptive behavior

Although the overall distribution of VABS composite scores was non-normal, distributions did not violate Shapiro-Wilk tests in each group separately (*p* ≥ .07). Therefore, one-way ANOVAs were used to compare clusters on VABS composite scores separately in each diagnostic group. VABS scores did not differ between clusters in the ASD sample, *F*(3, 113) = 2.04, *p* = .11. Among typically developing participants, the VABS composite did not differ between clusters, *F*(3, 65) = 1.48, *p* = .23.

#### Anxiety

CBCL DSM-oriented anxiety T-scores had a non-normal distribution, Shapiro-Wilk *W* = .63, *p* < .0001. Furthermore, autistic participants had significantly higher anxiety levels than typically developing participants, Wilcoxon-Mann-Whitney *W* = 6043.5, *n*_ASD_ = 126, *n*_TD_ = 75, *p* = .0003, δ = .28. Therefore, one-way non-parametric Kruskal-Wallis tests were used to compare clusters on anxiety scores separately in each diagnostic group. Anxiety levels did not differ between clusters among autistic participants, *H*(3) = 0.25, *p* = .97, or typically developing participants, *H*(3) = 0.97, *p* = .81.

### Cluster-based permutation correlation analyses

#### Caregiver-reported sensory symptoms

**Fig. 6:**
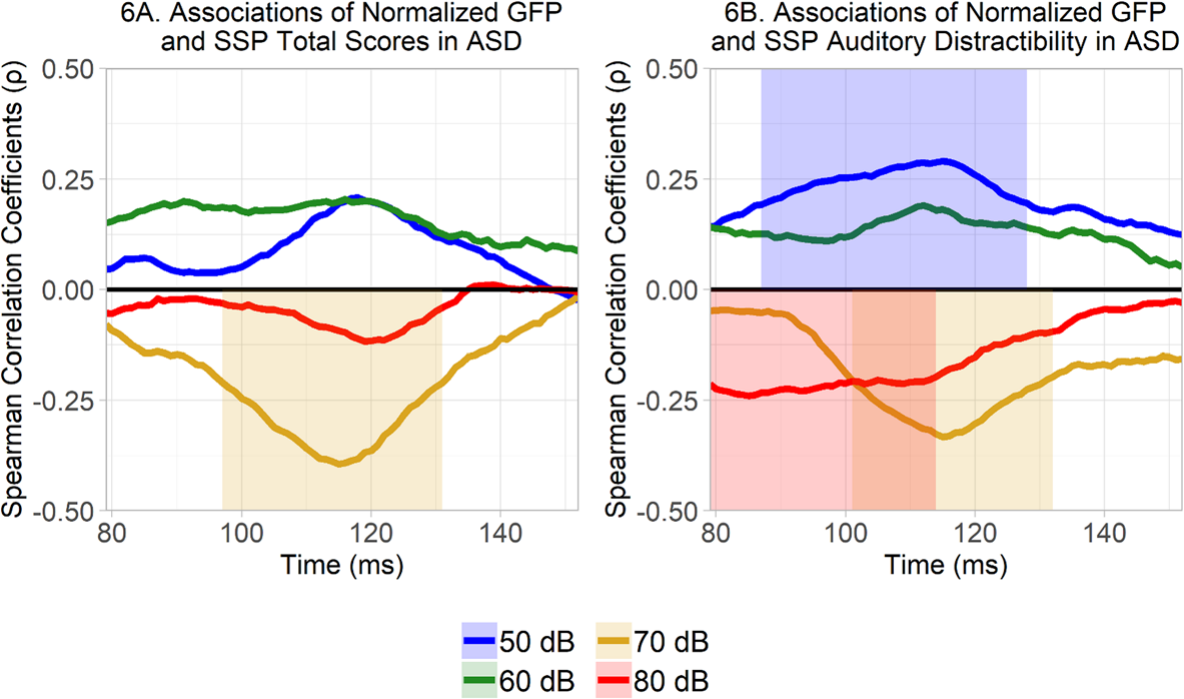
**a**, **b** Spearman’s correlation coefficients between normalized GFP in each condition, separately at each consecutive time-point, and other measured variables. Time windows with positive correlation effects in any loudness condition are highlighted above the zero-line, while time windows with negative correlation effects in any loudness condition are highlighted below the zero-line. Values at any given time point represent the Spearman’s correlation coefficient value at that time point. **a** Spearman’s correlation between normalized GFP and SSP total scores in ASD. **b** Spearman’s correlation between normalized GFP and SSP auditory distractibility in ASD

In ASD, there was a significant negative Spearman correlation between SSP total scores and normalized GFP to 70 dB sounds in a contiguous series of time points spanning between 97 and 131 ms, *p* = .009: that is, autistic participants with strong responses to 70 dB sounds in this approximate time period had, overall, more atypical caregiver-reported sensory processing features (Fig. 6a). There were no significant associations between SSP total scores and normalized GFP in any other loudness condition in ASD.

In ASD, there was a significant positive correlation between SSP auditory distractibility scores and normalized GFP to 50 dB sounds in a contiguous series of points spanning between 87 and 128 ms, *p* = .01 (Fig. 6b). There was no significant association between SSP auditory distractibility scores and normalized GFP to 60 dB sounds in ASD. However, there was a significant negative association between SSP auditory distractibility scores and normalized GFP to 70 dB sounds between 101 and 132 ms, *p* = .02. Furthermore, in ASD, there was a significant negative association between SSP auditory distractibility scores and normalized GFP to 80 dB sounds between 79 and 114 ms, *p* = .02. In other words, autistic participants with relatively weak responses to soft 50 dB sounds and relatively strong responses to louder 70 dB and 80 dB sounds were reported by caregivers to have more auditory distractibility problems.

#### Cognitive ability

In ASD, there were no significant associations between normalized GFP and MSEL DQ or MSEL NVDQ in any loudness condition or diagnostic group. Furthermore, in TD, there were no significant associations between normalized GFP and MSEL VDQ in any loudness condition or diagnostic group.

## Discussion

### Clustering and individual differences research with ERPs

This study demonstrates that it is possible to use the hierarchical clustering of electrophysiological data to group autistic and typically developing participants into clusters based on the loudness-dependency of their brain responses to auditory tones. As predicted by the first hypothesis, with normalized GFP as a dependent variable, there were robust differences in the pattern of loudness dependency between clusters. Visual inspection of the averaged normalized GFP across clusters (Fig. 3) as well as the waveforms in each cluster (Fig. 4) clearly reveals large, inter-cluster differences in the magnitude of the brain’s electrophysiological responses to stimuli of differing loudness. Thus, the clustering method was able to identify what we take to be meaningful differences in loudness-dependent response profiles. Indeed, it is important to note, as discussed further below, that the clusters defined on the basis of ERP responses differed from one another in other variables as well: notably, diagnostic group, performance on cognitive measures, and caregiver-reported sensory symptoms, which emphasizes the meaningfulness of the clusters. Interestingly, however, the clusters did not differ in the chronological ages of their participants, which suggests that developmental changes in auditory evoked responses do not affect the loudness-dependency of overall response strength in the time window of the present study.

Indeed, it is not clear at this point what neural mechanism might be responsible for these individual differences in loudness-dependent neural responses. It has been suggested that individual differences in intensity-dependent N1/P2 auditory responses in adults reflect variation in serotonergic neurotransmission [82]. However, even in adults, empirical evidence regarding this hypothesis is mixed, as is evidence regarding associations between loudness-dependent auditory responses and dopamine [83]. Given the excitation-inhibition balance hypothesis of autism [84, 85], we are intrigued by the possibility that loudness-dependent responses might relate to neural excitation and inhibition.

### Overlap between diagnostic groups

As predicted by the second hypothesis, there was considerable overlap between the diagnostic groups in the clusters defined in the present analysis. Although the proportions of autistic and typically developing participants did differ significantly across clusters, these differences were modest. All clusters contained substantial numbers of both autistic and typically developing participants. This overlap across diagnostic groups appears to emphasize the complexity of the individual differences in neural processes that underlie both the autistic and typically developing auditory processing.

### Description of clusters and associations with other measures

The results of the present study are not consistent with the third hypothesis, which predicted that typically developing participants would be more likely to fall into clusters characterized by a pattern of neural responses increasing in strength monotonically as the loudness of the tones increased, while autistic participants might be more likely to appear in clusters with either non-monotonic patterns or unexpectedly strong increases in neural response strength with intensity. As can be seen in Fig. 3, the only groups which approximate monotonicity are C1 and C2. However, the only cluster in the observed data that trended to have a disproportionate number of typically developing participants was not C1 but C3, and contrary to prediction, C3’s participants actually displayed an unexpectedly strong, non-monotonic responses to softer, 60 dB sounds.

On the other hand, C1 had a disproportionately large number of autistic participants: this cluster exhibited a response to 70 dB sounds that was stronger than that found in any other cluster, as well as a stronger 60 dB response than C2 or C4. To further characterize this cluster, one can turn to the various measures and assessments collected in the APP. Curiously, although autistic participants were more likely to fall into C1 than other clusters, autistic participants in C1 did not appear to have more conspicuously atypical phenotypes than autistic participants in other clusters. Indeed, the data reveal the opposite. Among autistic participants, full-scale and nonverbal DQ on the MSEL did significantly differ between clusters, and a similar effect of verbal DQ fell just short of statistical significance. While results of follow-up tests no longer attained statistical significance after a robust correction for multiple comparisons, trends suggest that the significant effects were driven by higher scores in C1. Replication and further research are needed to confirm and fully understand these findings, in part because, as noted in supplementary materials, a subgroup of participants from C1 would often be reclassified into C2 when subsamples were repeatedly drawn and re-clustered. However, it is interesting to note that, in ASD, there were no significant continuous associations between MSEL scores and normalized GFP in any loudness condition. This perhaps suggests that the high cognitive abilities of autistic participants in C1 are driven not by the strength of their responses to any given intensity (such as their strong 70 dB response) but by their overall pattern of response monotonicity across loudness conditions.

Meanwhile, the other approximately monotonic cluster, C2, is characterized primarily by an extremely strong response to the very loudest, 80 dB stimuli. Autistic and typically developing participants appeared to have roughly equal probabilities of being classified into C2, but membership in C2 appeared to be linked to sensory behaviors. The fourth hypothesis suggested that auditory sensory behaviors would be more typical in clusters characterized by a pattern of neural responses increasing in strength in a monotonic fashion similar to the typical grand-average pattern, but C2 does not appear to meet these criteria. Responses appear roughly monotonic, but from visual inspection, the strength of the 80 dB response seems to greatly exceed the overall grand-average. Indeed, among autistic participants, exploratory analyses indicated that caregiver-reported problems with auditory distractibility were significantly greater in C2 than C4. Furthermore, although the post-hoc effect was no longer significant after correction for multiple comparisons, auditory distractibility problems strongly trended towards being greater among autistic participants in C2 than C3. Similarly, continuous associations suggested that auditory distractibility problems were greater in autistic participants exhibiting a pattern of relative neural hyper-responsiveness to loud 70 dB and 80 dB sounds and relative neural hypo-responsiveness to soft 50 dB sounds.

These findings should be interpreted with care owing to the ambiguity of the “auditory distractibility” factor; for example, it seems possible to interpret the SSP items loading on this factor (“Is distracted or has trouble functioning if there is a lot of noise around,” “Can’t work with background noise,” and “Has trouble completing tasks when the radio is on”) as signs of general difficulty functioning in noisy environments rather than distractibility per se. Thus, neural over-responsiveness to loud sounds in at least a subgroup of autistic participants appears to be related to abnormal behavior—distractibility and/or difficulty functioning—in noisy environments. The possibility that these results could be related to hyperacusis, which has been observed in ASD [5–8], appears intriguing. However, such an interpretation must be considered tentative as the present study does not assess loudness discomfort levels. Furthermore, similar neural responses in TD participants from C2 were not linked to any pattern of caregiver-reported auditory sensory behaviors, indicating that the presence of these relatively stronger responses to loud sounds is not a sufficient condition for auditory sensory sensitivity.

Verbal cognitive ability among typically developing participants was lower in C2 than C4, with similar but nonsignificant trends for VDQ to be lower in C2 than C1 and C3. However, although C2 appeared to be characterized by abnormally strong responses to 80 dB sounds, the mean VDQ differences between C2 and other clusters in TD were surprisingly not accompanied by significant continuous associations between normalized GFP to 80 dB sounds and MSEL VDQ in TD, complicating their interpretation. Replication and further research appear necessary before this effect can be confirmed and understood.

## Limitations

The present study has a number of strengths. It is based on a large, well-characterized sample of autistic and typically developing participants drawn from a relatively narrow chronological age range. Many trials were collected from each participant, and data were subjected to an intensive processing pipeline, allowing us to have considerable confidence in individuals’ observed average responses in each loudness condition. Furthermore, the focus on loudness-dependent normalized responses allows the analysis to circumvent individual differences in biophysical factors such as skull thickness, while the use of GFP gives the study an overall metric of neural response strength that avoids the need for a priori decisions about analyzing particular components or electrode sites.

Along with these strengths, the present study has limitations. One is the use of a brief, 38-item caregiver report of sensory behaviors that was not originally designed for use in autism. Future studies could use other parent-report measures or, when participants’ verbal abilities permit, self-reports. It should also be noted that our analyses examined a large number of variables like MSEL DQ and SSP sensory behaviors in an exploratory manner.

Another limitation of the present study is that there is naturally a loss of information in any procedure (such as the clustering procedure employed here) that reduces the complexity of the data. To address this limitation, future analyses will cluster participants based on the latency of ERP responses. The present analysis also ignores the topographic distribution of neural responses. The authors plan in future analyses to define clusters not only based on timing but also on the topographic distribution of neural responses over pre-defined scalp regions.

We also acknowledge that the present study did not involve the collection of hearing acuity measures. This reflected the difficulty of obtaining reliable estimates of hearing acuity in young children, especially in groups with diverse language and cognitive abilities. However, prior research suggests that there is variability in hearing acuity within ASD [6, 7]. While the within-participants normalization of GFP across loudness intensities in the present study could offer some protection against any individual differences in overall hearing acuity, this procedure does not account for non-linearities in the relations among GFP strength, stimulus intensity, and hearing thresholds. It is thus unclear how hearing acuity might relate to the individual differences observed in this study.

More generally, one might argue that the ultimate goal of projects examining neural sensory responses—such as the present study—is to gather information related to the internal sensory experience of an individual [86]. However, the cortical auditory response from ~80–150 ms post-stimulus onset is only one contributor to these internal sensory experiences, which are likely to be emergent properties of many neural processes. Thus, two individuals with cortical neural responses of equal strength in the 80–150 ms window might nevertheless have different sensory experiences. Furthermore, one must consider that brief auditory complex tones differing in loudness are only one of the numerous types of stimuli that exist in the auditory domain alone. This should not be interpreted as an argument against electrophysiology and other neuroimaging techniques, but as a buttress to existing suggestions (e.g., by [21]) that multiple methods should be employed to understand individual differences in sensory experience.

## Conclusions

We believe that this study demonstrates that clustering can be used to meaningfully describe the variability of neurophysiological event-related responses in sufficiently large datasets and that normalization of responses across conditions may be valuable in focusing on neural variations in response strength across these conditions. As confirmed by chi-square analysis, autistic and typically developing participants were distributed significantly differentially across clusters. Additional analyses suggested that participants in a cluster characterized by neural hyper-responsiveness to loud sounds exhibited auditory distractibility/filtering problems; similarly, in continuous associations, relative neural hyper-responsiveness to loud sounds was associated with auditory distractibility. Furthermore, autistic participants in a cluster characterized by relatively monotonic responses seemed to exhibit higher total cognitive abilities, although post-hoc comparisons fell short of significance after correction for multiple comparisons. We also observed the effects of cognitive ability in typical development, although these findings may require replication and further research to be understood. Overall, however, the existence of relationships between cluster membership and other variables suggests that the present clusters constitute and describe meaningful variability, though we draw no conclusion to the effect that the clusters represent categorically discrete populations. We suggest that clustering analyses similar to those used in this study may be valuable tools in future research describing additional dimensions of neural heterogeneity.

## Supplementary information

**Additional file 1.** Supporting Information.

## Data Availability

The data from which classifications and statistical results in the present paper were derived will be made available in an appropriate repository—either OSF or NDAR subsequent to publication.

## Abbreviations

APP: Autism Phenome Project ASD Autism spectrum development ASR Adjusted standardized residual CBCL Childhood Behavior Checklist DQ Developmental quotient EEG Electroencephalography RP Event-related potential GFP Global field power MSEL Mullen Scales of Early Learning NVDQ Non-Verbal Developmental Quotient SMART Semi-Automatic Artifact Removal Tool SOBI Second-Order Blind source Identification SSP Short Sensory Profile TD Typical development VABS Vineland Adaptive Behavior Scales VDQ Verbal Developmental Quotient

## Footnotes

1 Note on terminology: In light of research [1] suggesting that the majority of autistic-people prefer identity-first language (e.g., “autistic”) to person-first language (e.g., “person with autism”), the authors of the present paper have chosen to use identity-first language. Furthermore, since this study [1] also found that only a minority of autistic people endorsed the use of the terms “disorder” or “condition” to describe autism, and out of a desire to avoid unnecessarily embedding value judgements into research terminology, the authors have decided to use “autism spectrum development” as an alternative.

2 This statistical test is often referred to as a “cluster-based permutation test.” However, we have chosen to use the term “contiguity-based” to avoid confusion with the hierarchical clustering analysis also employed in this paper.
