# Supplementary material for: Defining clusters of young autistic and typically developing children based on loudness-dependent auditory electrophysiological responses
Source: Mol Autism. 2020 Jun 15;11:48. doi: 10.1186/s13229-020-00352-3 (PMC7294610; doi:10.1186/s13229-020-00352-3)
Supplement: Supplementary file 1 — Additional file 1. Supporting Information. [file 13229_2020_352_MOESM1_ESM.docx]

# Supporting Information

## Supplementary Methods

### Resampling Analysis.

As an initial examination of whether participants could be reclassified in the same manner as obtained in the clustering analysis presented in the main text, subsamples of 80% of all participants in the present study were resampled 1000 times with replacement. Each of these subsamples were then clustered using Ward’s method (as described in the main text) with the number of clusters fixed to four. We then calculated the average probabilities that individual participants from each of these clusters would be classified together with other participants that had also been assigned to their cluster in the main analysis, as well as the average probability that participants would be classified together with participants that had been assigned to different clusters in the main analysis.

This procedure was then repeated with subsamples of 50% of all participants, which were drawn 1000 times and clustered with Ward’s method so that the probabilities of reassignment alongside the same participants could be calculated as described above. We conducted this additional analysis with 50% subsamples because we wished to verify that the replicability of results obtained with 80% subsamples – arguably likely to be fairly similar to the full dataset – would generalize to smaller subsamples.

### Within-Cluster SSP Comparisons.

Given the non-normal distributions of SSP scores noted in the main text, Wilcoxon-Mann-Whitney tests were used to compare whether autistic and typically-developing participants in each cluster differed in their SSP total scores and auditory subscores. Cliff δ [93] was used as a measure of effect size and confidence intervals are reported. Note that no multiple comparison corrections were applied.

### Number of Trials

To rule out the possibility that the number of trials could have differed across clusters, mixed ANOVA was used to examine effects of cluster and diagnostic group on both the original number of recorded trials and the final number of trials included in the analysis.

## Supplementary Results

### 80% Subsample Resampling Analysis.

For all participants, average probability of being re-classified with other participants from their original cluster was substantially greater than probability of being re-classified with participants from other original clusters (Table S2; *Figure S1*). However, a small subgroup of participants assigned to C1 in the original analysis appear to move back and forth between C1 and C2 in the resampled clusters. These participants do have somewhat weaker 70 dB and somewhat stronger 80 dB responses than the rest of the participants in C1, but they do not have the *disproportionately* strong 80 dB response characterizing C2. For this reason, and because of their assignment to C1 in the original analysis with the full sample, they were retained in C1.^[[1]](#footnote-1)^

### 50% Subsample Resampling Analysis

In the event, the results of the 50% subsample analysis proved quite similar to the results of the 80% resampling analysis. In these re-samplings, participants continued to be more likely to be classified into clusters alongside other participants from their original clusters than to be classified alongside participants from other clusters in the original dataset (Table S3). Likewise, visual inspection suggested that patterns of cluster reassignment were for the most part relatively stable, again with the exception that certain participants from C1 would appear to move back and forth between C1 and C2 (*Figure S2*).

### Within-Cluster SSP Comparisons

SSP total and auditory subscore differences between autistic and typically-developing participants within specific clusters did not always exceed the threshold for statistical significance, but many of autistic participants’ scores were significantly lower than those of typically-developing participants (Table S4). Auditory Distractibility scores were significantly lower (more atypical) in ASD in all clusters save C4 (and even in C4 the confidence interval of δ did not include zero). Hyporesponsiveness to Speech and total scores significantly differed across diagnostic groups in all clusters. Noise Distress scores significantly differed between diagnostic groups in C2. This suggests—unsurprisingly given that the present analysis only compared the relative strengths of neural responses to sounds varying along a single dimension, namely loudness, in the approximate latency range of the P1 ERP component—that there is meaningful variability in sensory processing patterns not captured by the present analysis, pointing to a need for further research exploring additional dimensions of sensory heterogeneity.

### Number of Trials.

In the mixed ANOVA with the total number of trials in the original recordings as the dependent variable, there was no main effect of cluster, *F*(3,205) = 0.08, *p* = .97, $\eta_{G}^{2}$ = .00, nor a main effect of diagnostic group, *F*(1,205) = 1.06, *p* = .31, $\eta_{G}^{2}$ = .01, nor any interaction between clusters and diagnostic group, *F*(3,205) = 0.07, *p* = .98, $\eta_{G}^{2}$ = .00. Furthermore, the ANOVA examining effects of cluster and diagnostic group on the total number of trials retained after data processing also found neither a main effect of cluster, *F*(3,205) = 0.12, *p* = .95, $\eta_{G}^{2}$ = .00, nor an interaction between cluster and diagnostic group, *F*(3,205) = 0.48, *p* = .70, $\eta_{G}^{2}$ = .01. However, there was a main effect of diagnostic group, *F*(1,205) = 7.35, *p* = .01, $\eta_{G}^{2}$ = .03, on the number of trials retained after processing. This pattern of results suggests that data quality was unsurprisingly slightly poorer in the ASD group, apparently leading to rejection of additional trials in data processing, but this difference did not interact with cluster assignment, which we believe offers support to the validity of our clustering results.

## Supplementary Tables

| Table S1. *SSP total scores and auditory subscores for typically-developing participants by cluster.* | | | | | | | | | | | | |
| --- | --- | --- | --- | --- | --- | --- | --- | --- | --- | --- | --- | --- |
|  | Cluster Means (Standard Deviations) | | | | Kruskal-Wallis Test | | Cliff’s δ^1^ | | | | | |
|  | C1 | C2 | C3 | C4 | *H*(3) | *p* | C1, C2 | C1, C3 | C1, C4 | C2, C3 | C2, C4 | C3, C4 |
| Auditory Distractibility | 13.93 (1.00) | 13.17 (1.64) | 14.00 (1.36) | 14.07 (0.83) | 2.92 | .41 | .24 | –.13 | –.08 | –.29 | –.27 | .10 |
| Hyporesponsiv-eness to Speech | 8.29 (1.59) | 7.50 (2.02) | 8.27 (1.34) | 8.36 (1.22) | 1.62 | .65 | .23 | .03 | .02 | –.22 | –.26 | .03 |
| Noise Distress | 7.93 (1.90) | 8.17 (1.85) | 8.42 (1.77) | 8.36 (1.39) | 0.85 | .84 | –.08 | –.17 | –.09 | –.08 | –.02 | .09 |
| Total SSP Score | 165.71 (11.80) | 164.25 (10.96) | 170.12 (16.08) | 167.23 (11.34) | 3.70 | .30 | .08 | –.26 | –.07 | –.32 | –.15 | –.24 |
| ^1^ Values of δ are flagged with * if the corresponding Wilcoxon-Mann-Whitney *p* value is < .05 after Bonferroni-Holm correction for six comparisons. | | | | | | | | | | | | |

| Table S2. *Averaged probabilities from the 80% subsample resampling analysis of individual participants (broken down based on assignment to clusters in the original analysis) being classified together with individual participants that were, or were not, assigned to their cluster in the original analysis.* | | | | |
| --- | --- | --- | --- | --- |
| Original Cluster of Participant | C1 | C2 | C3 | C4 |
| Averaged probability of being re-clustered with participants that had been assigned to the same cluster in the original analysis | .48 | .61 | .61 | .67 |
| Averaged probability of being clustered with participants that had been assigned to other clusters in the original analysis | .20 | .21 | .13 | .17 |

| Table S3. *Averaged probabilities from the 50% subsample resampling analysis of individual participants (broken down based on assignment to clusters in the original analysis) being classified together with individual participants that were, or were not, assigned to their cluster in the original analysis.* | | | | |
| --- | --- | --- | --- | --- |
| Original Cluster of Participant | C1 | C2 | C3 | C4 |
| Averaged probability of being re-clustered with participants that had been assigned to the same cluster in the original analysis | .47 | .54 | .59 | .63 |
| Averaged probability of being clustered with participants that had been assigned to other clusters in the original analysis | .22 | .21 | .16 | .19 |

| Table S4. *Statistical comparisons of total SSP scores and auditory subscores across diagnostic groups in each cluster.* | | | | | | | | |
| --- | --- | --- | --- | --- | --- | --- | --- | --- |
|  | C1 | | C2 | | C3 | | C4 | |
|  | *p* | Cliff’s δ  (95% CI) | *p* | Cliff’s δ  (95% CI) | *p* | Cliff’s δ  (95% CI) | *p* | Cliff’s δ  (95% CI) |
| Auditory Distractibility | .0001 | –.69  (–.84, –.44) | .002 | –.65  (–.85, –.29) | .0002 | –.58  (–.78, –.28) | .05 | –.39  (–.68, –.002) |
| Hyporesponsiv-eness to Speech | <.0001 | –.87  (–.99, –.15) | .0006 | –.72  (–.89, –.38) | <.0001 | –.92  (–.99, –.27) | <.0001 | –.85  (–.96, –.54) |
| Noise Distress | .34 | –.17  (–.48, .18) | .03 | –.46  (–.73, –.05) | .42 | –.13  (–.42, .19) | .32 | –.20  (–.54, .20) |
| Total Score | <.0001 | –.81  (–.95, –.41) | .0009 | –.73  (–.91, –.31) | <.0001 | –.73  (–.90, –.35) | .0004 | –.76  (–.91, –.42) |
| P*-values are based on Wilcoxon-Mann-Whitney tests. C1 contains 39-42 autistic and 14 typically-developing participants with complete data on the different SSP factors and total score, C2 contains 18-21 and 12 participants respectively, C3 contains 23-26 and 26 respectively, and C4 contains 19 and 13-14 respectively. (Note that mean scores and standard deviations on each scale are presented, separated by diagnostic group, in Table 4 of the main text and in Table S1.)* | | | | | | | | |

## Supplementary Figures


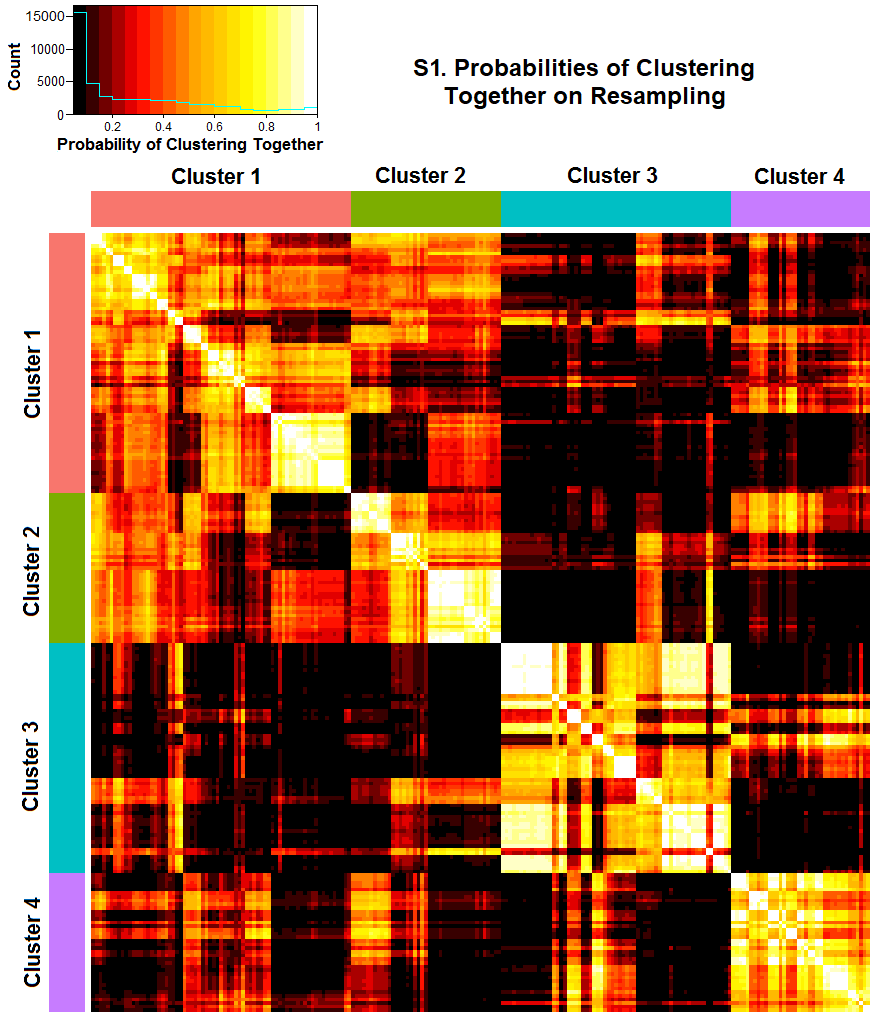


*Figure S1*. Results of a resampling analysis in which 80% of participants were sampled 1000 times with replacement and clustered using Ward’s method as described above. Values in the heatmap above are probabilities of each participant (represented as a row/column pair) clustering with every other participant. C3 and C4 appear highly stable, but a small subgroup of participants in the uppermost left corner (in C1) appear to move back and forth between C1 and C4. These participants do have somewhat weaker 70 dB and somewhat stronger 80 dB responses than the rest of the participants in C1, but they do not have the *disproportionately* strong 80 dB response characterizing C2. For this reason, they were retained in C1.


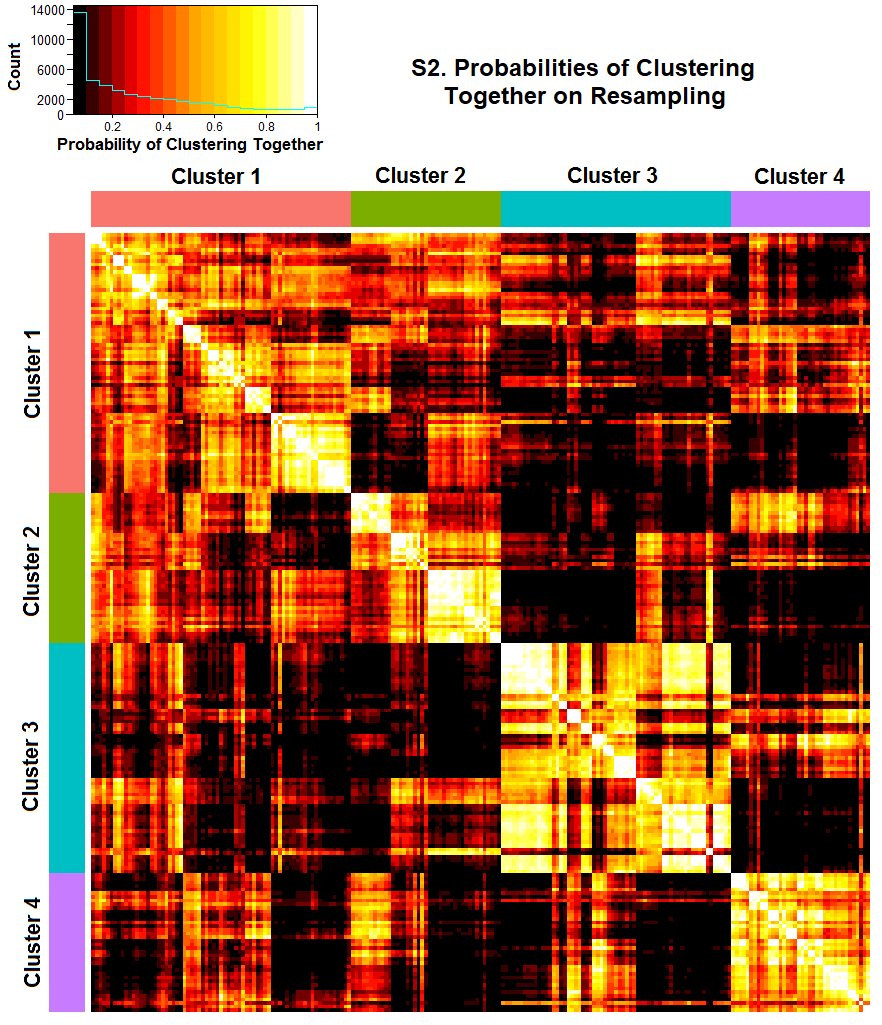
*Figure S2*. Results of a resampling analysis in which 50% of participants were sampled 1000 times with replacement and clustered using Ward’s method as described above. Values in the heatmap above are probabilities of each participant (represented as a row/column pair) clustering with every other participant. As with the 80% resamples analysis, C3 and C4 appear highly stable, but a small subgroup of participants in the uppermost left corner (in C1) appear to move back and forth between C1 and C4. These participants do have somewhat weaker 70 dB and somewhat stronger 80 dB responses than the rest of the participants in C1, but they do not have the *disproportionately* strong 80 dB response characterizing C2. For this reason, they were retained in C1.


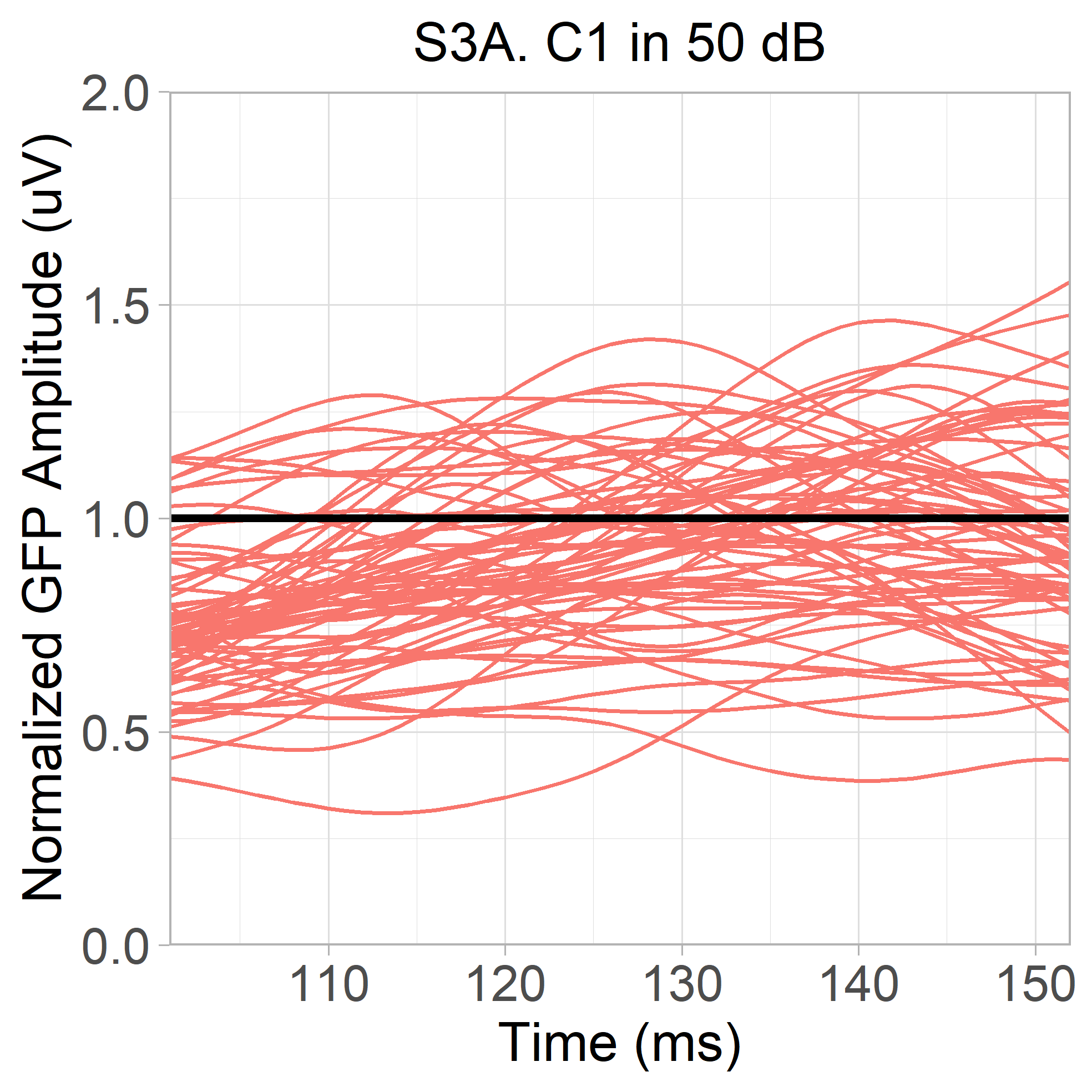

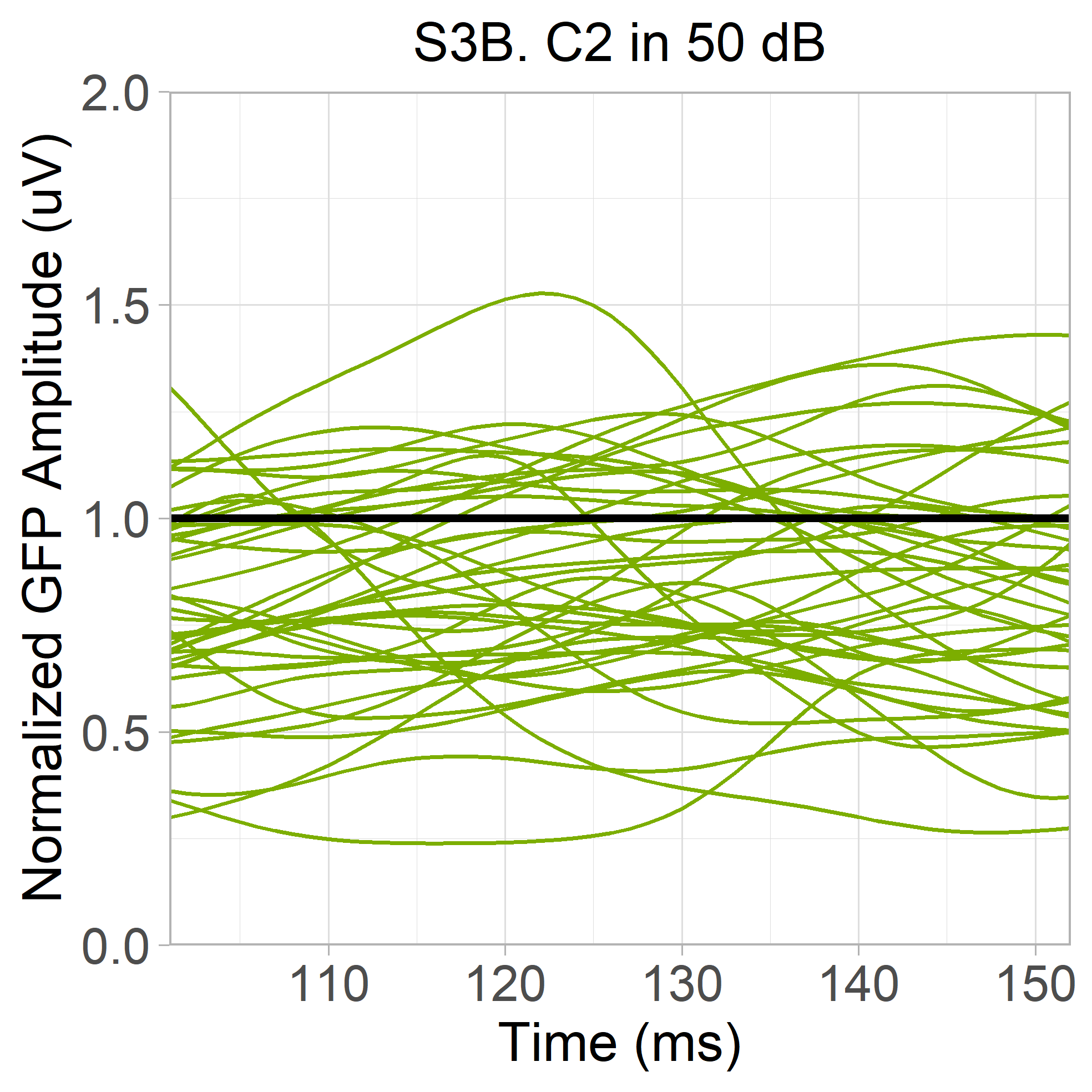


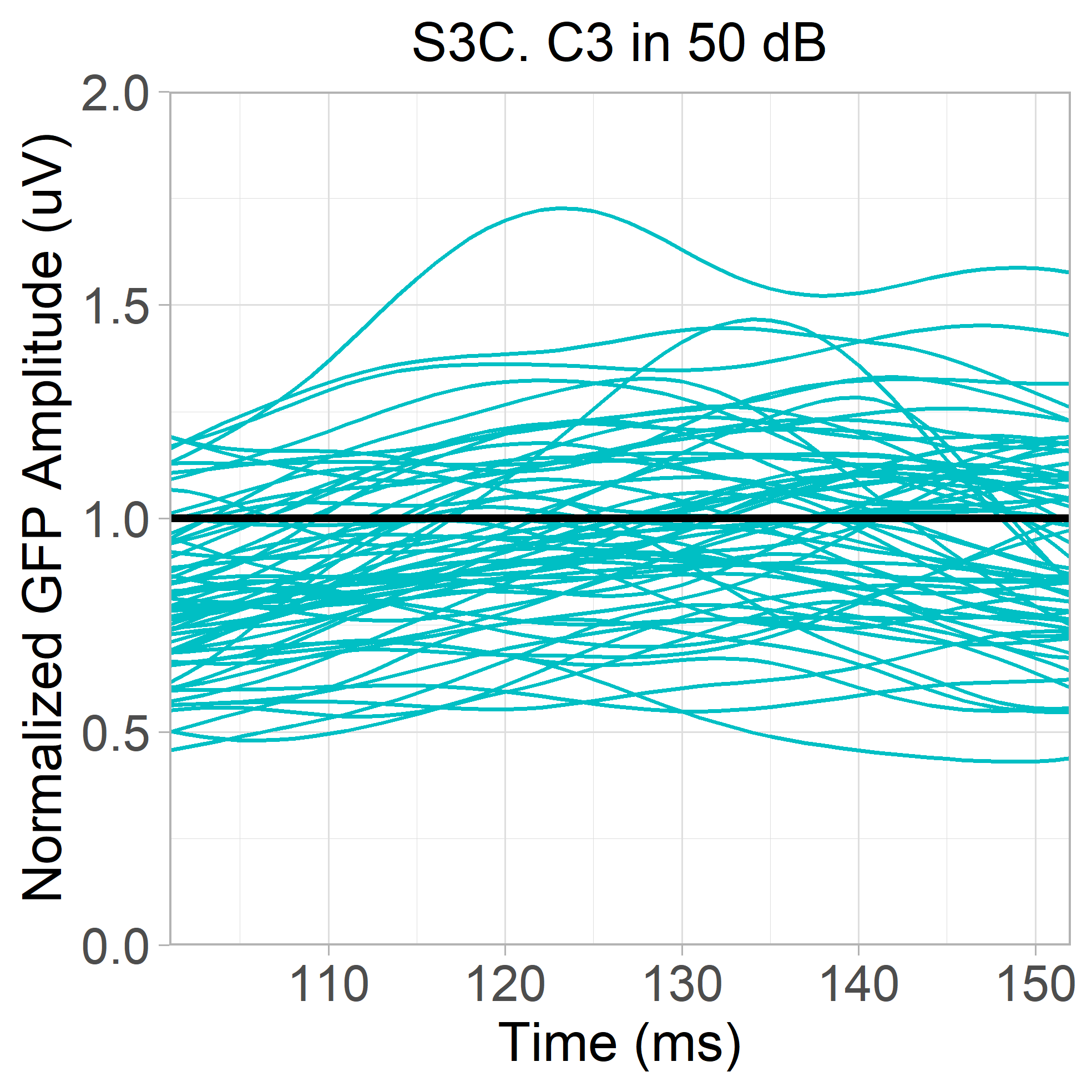

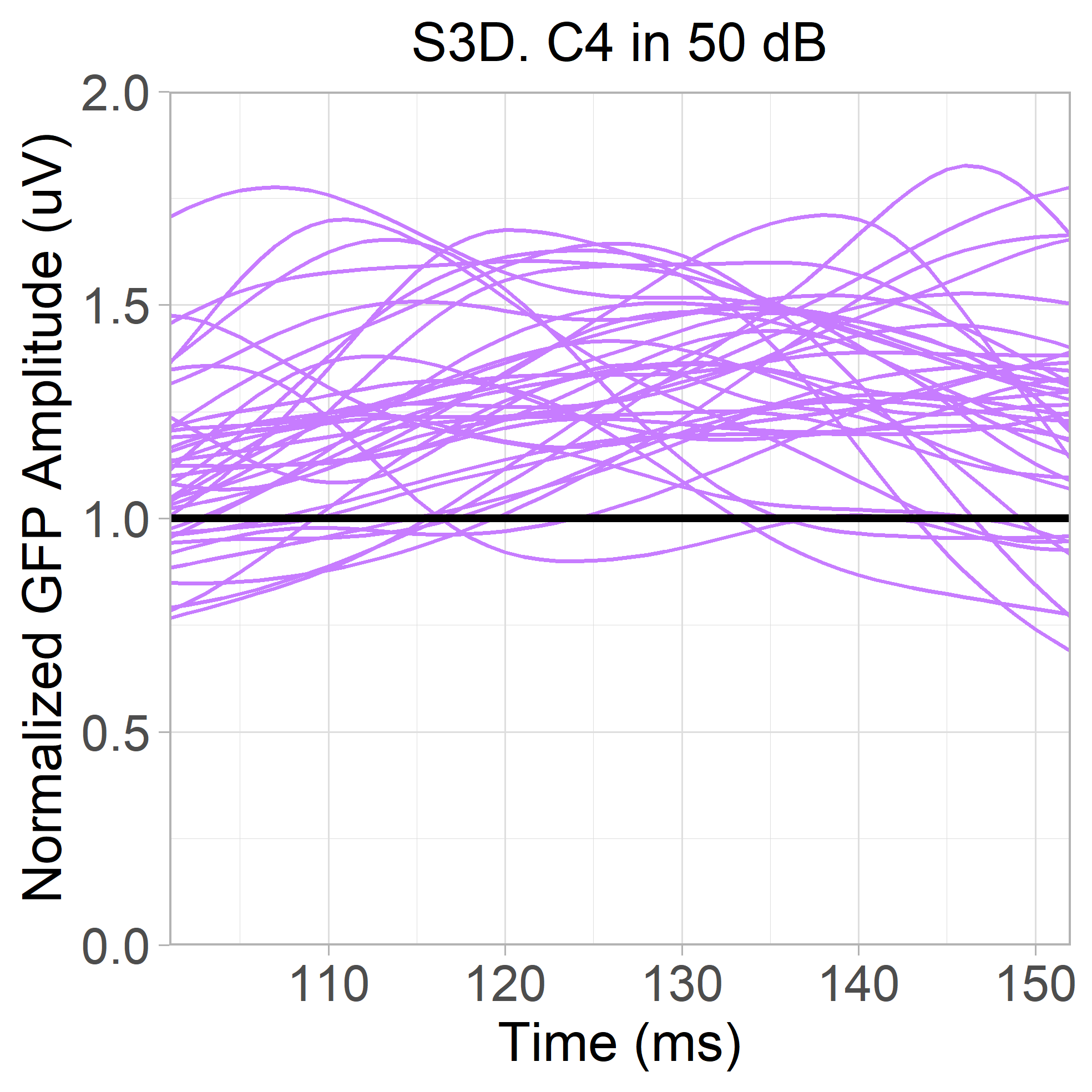


*Figure S3*. Spaghetti plots of normalized GFP waveforms from each participant in the 50 dB condition (101 – 152ms). Colours represent clusters. ***A.*** Responses from C1, containing 53 autistic and 18 typically-developing participants. ***B.*** Responses from C2, containing 24 autistic and 17 typically-developing participants. ***C*.** Responses from C3, containing 32 autistic and 31 typically-developing participants. ***D.*** Responses from C4, containing 23 autistic and 15 typically-developing participants.


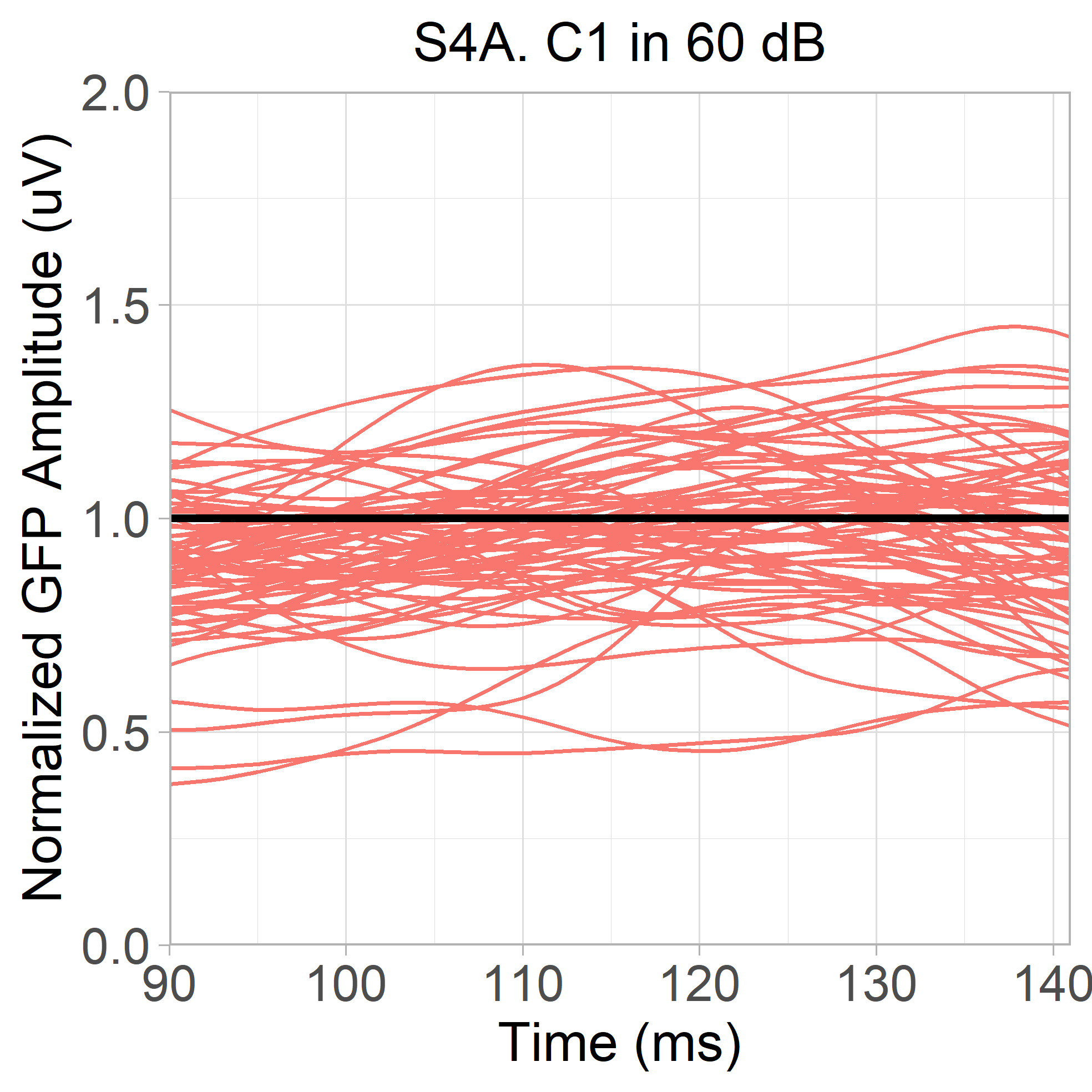

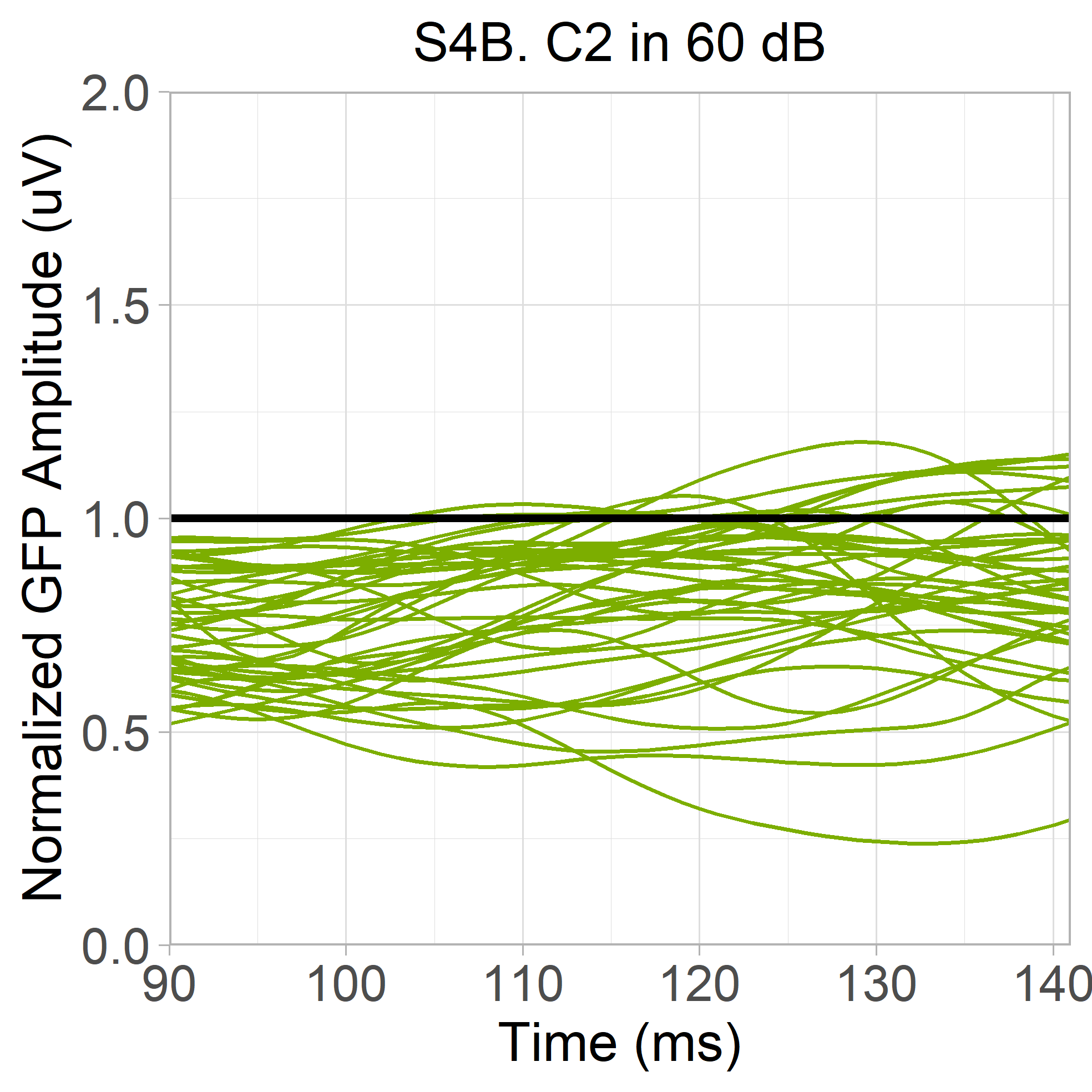


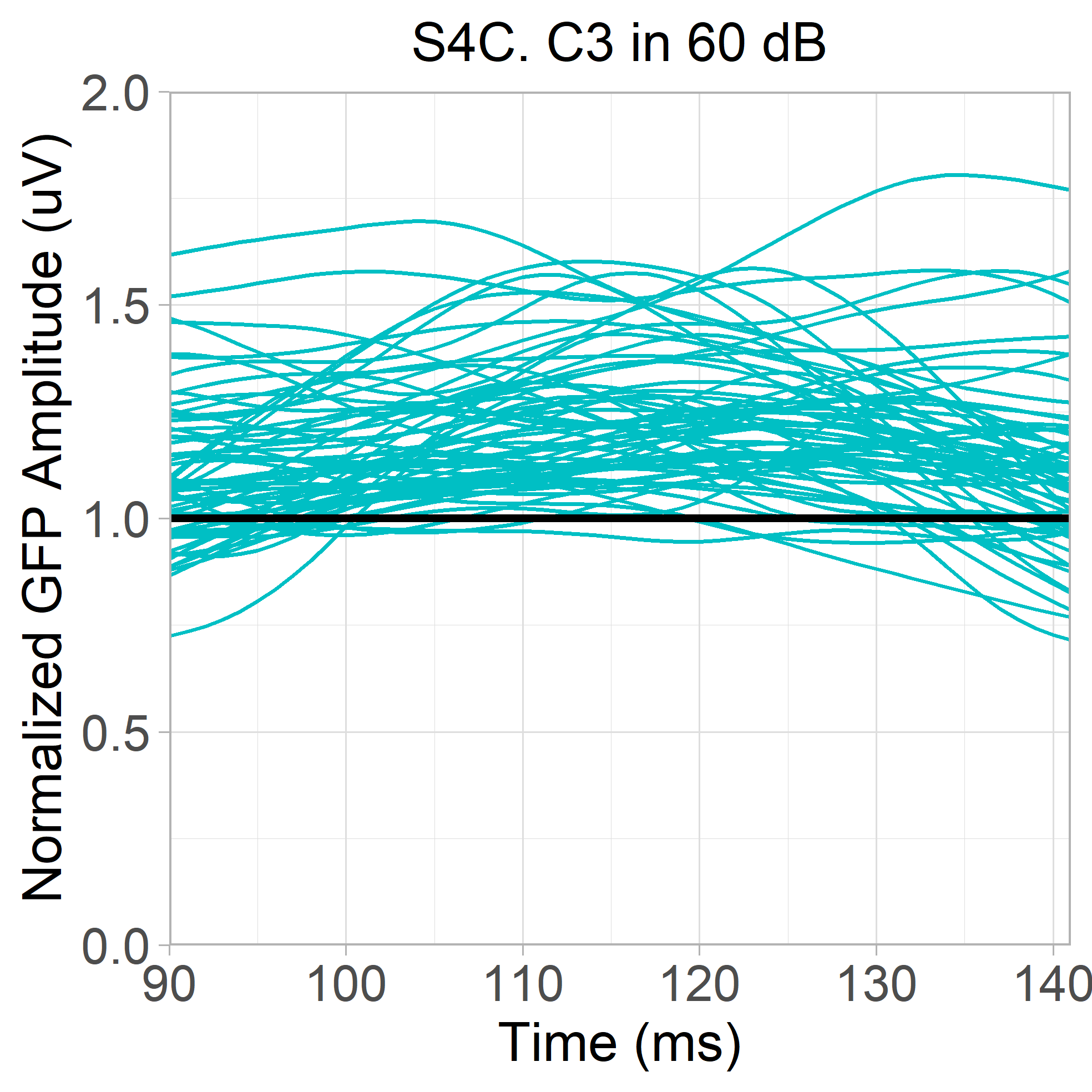

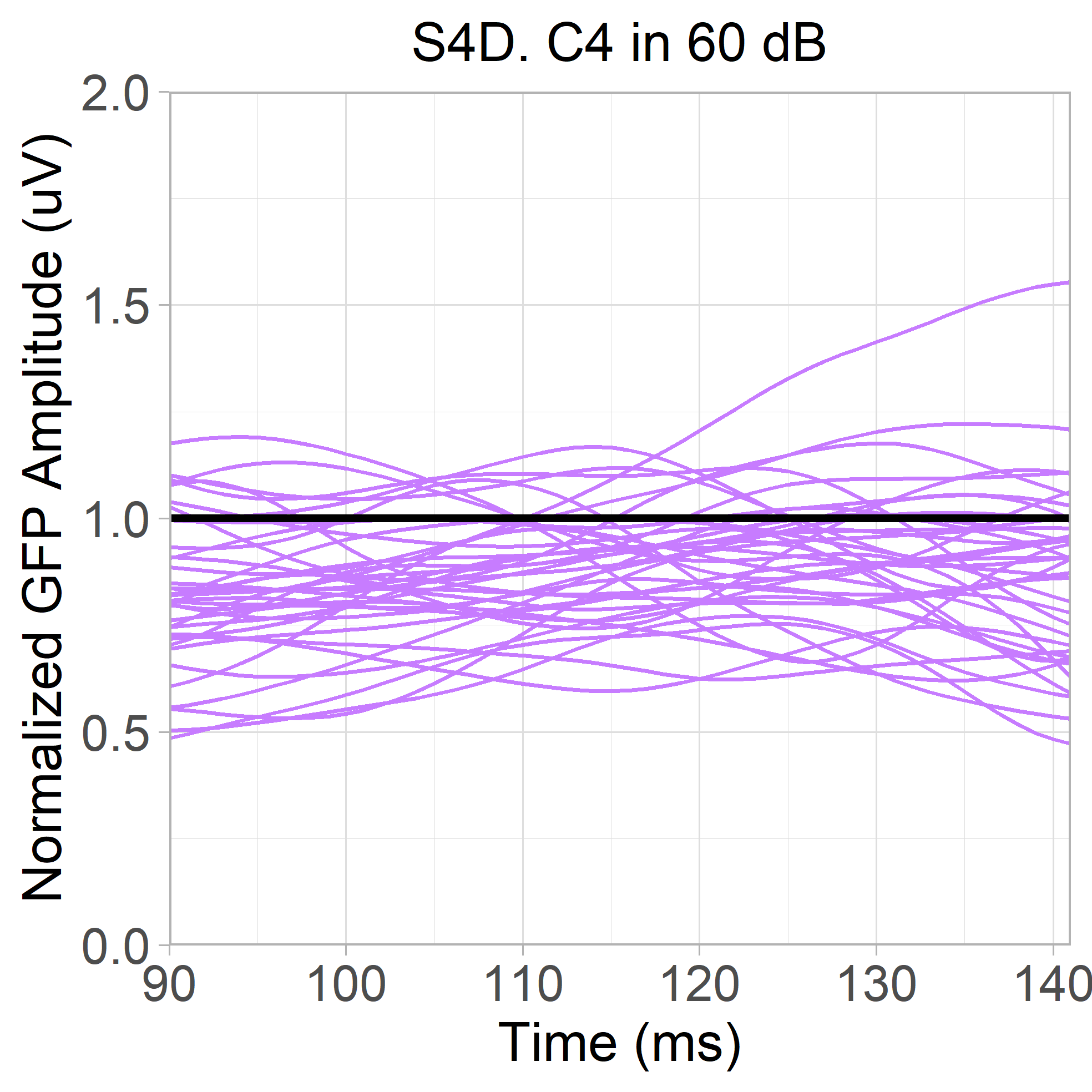


*Figure S4*. Spaghetti plot of normalized GFP waveforms from each participant in the 60 dB condition (90 – 141ms). Colours represent clusters. ***A.*** Responses from C1, containing 53 autistic and 18 typically-developing participants. ***B.*** Responses from C2, containing 24 autistic and 17 typically-developing participants. ***C*.** Responses from C3, containing 32 autistic and 31 typically-developing participants. ***D.*** Responses from C4, containing 23 autistic and 15 typically-developing participants.


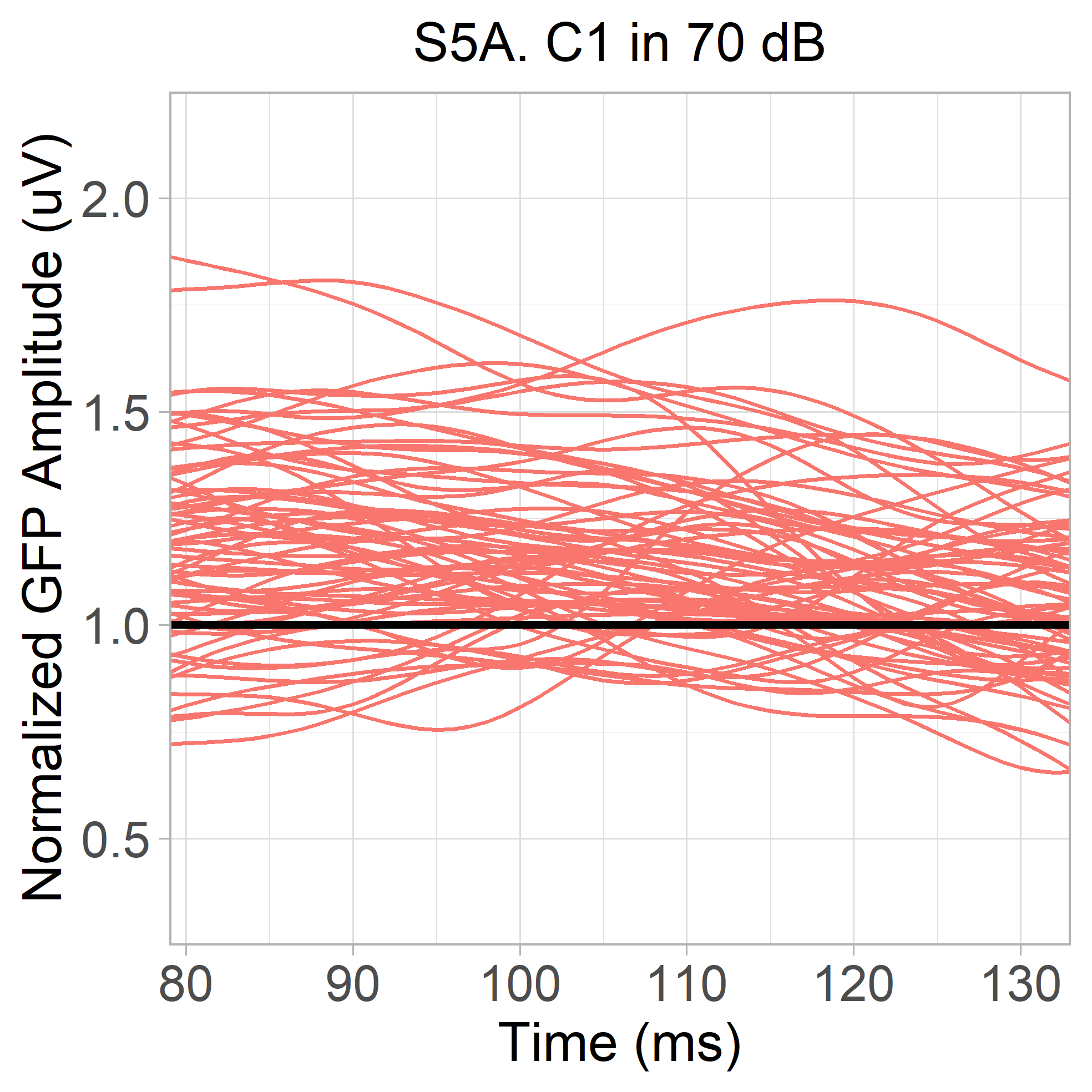

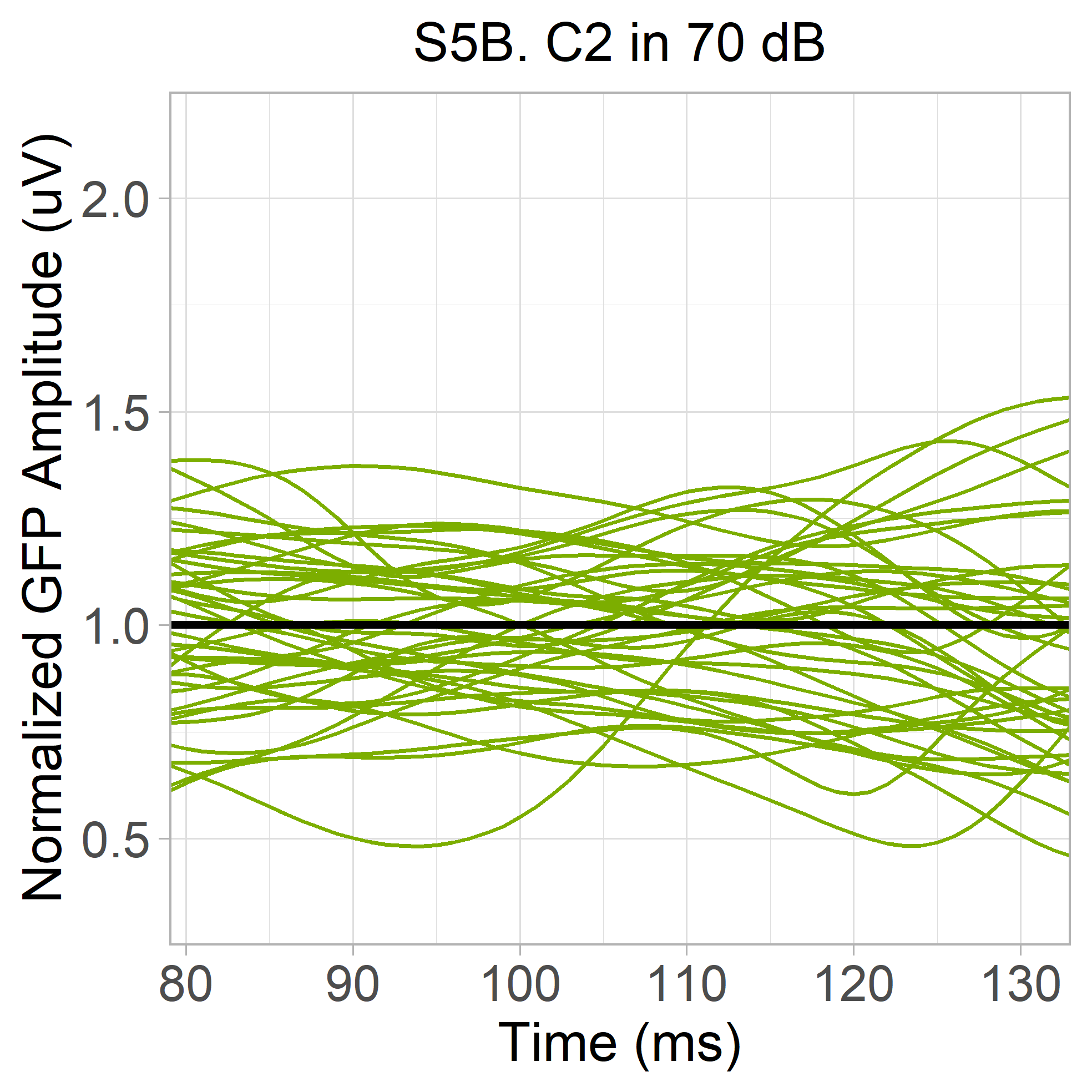


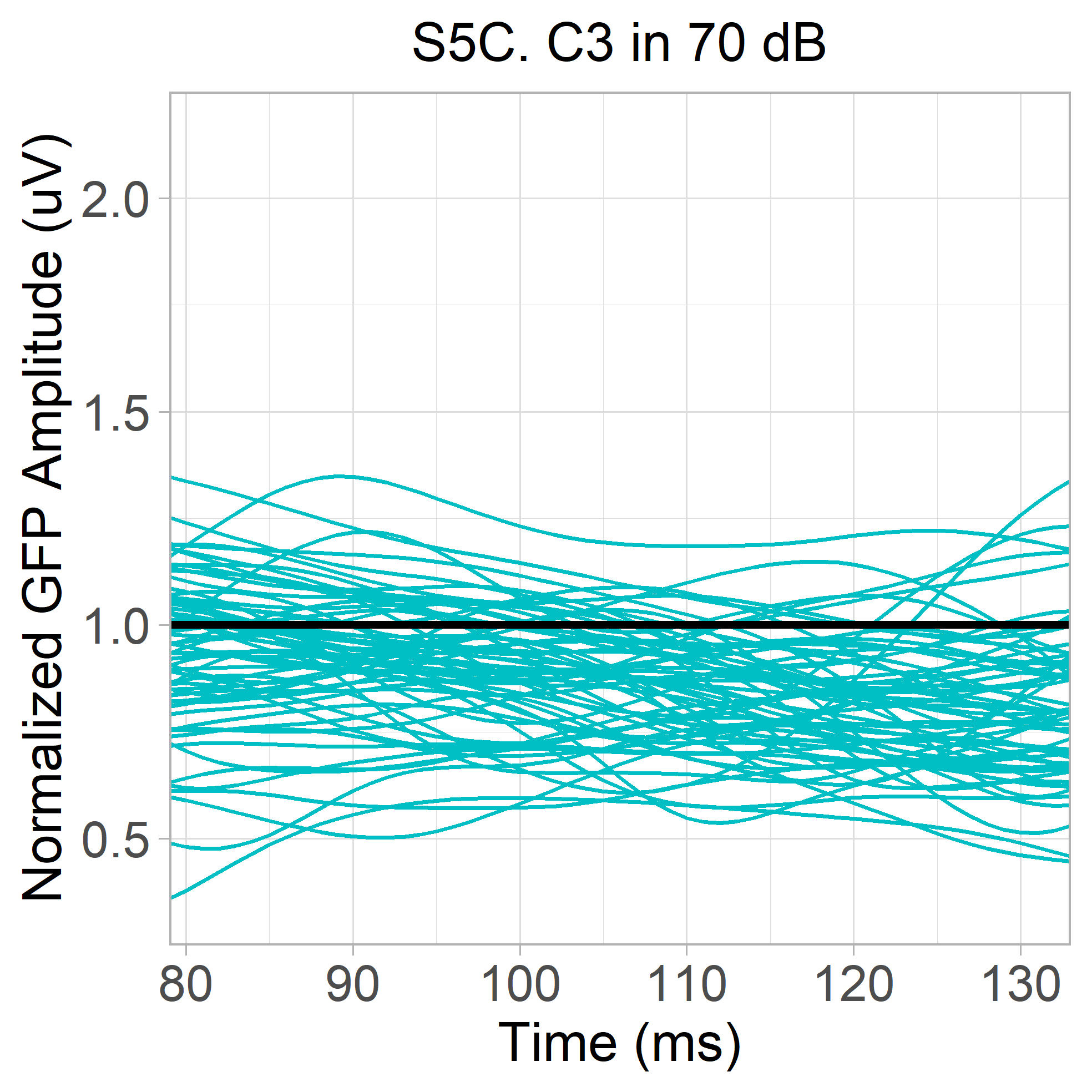

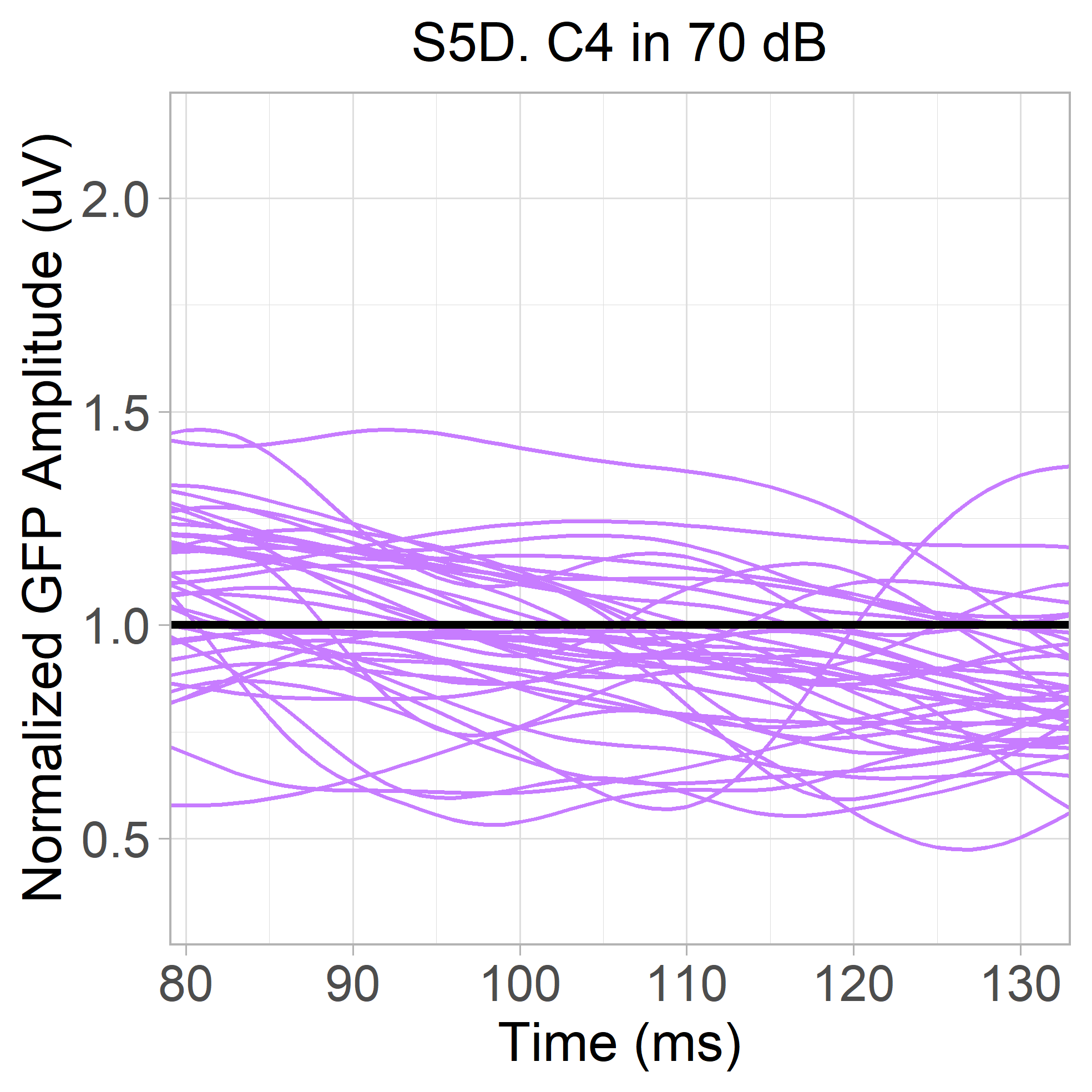


*Figure S5*. Spaghetti plot of normalized GFP waveforms from each participant in the 70 dB condition (79 – 133ms). Colours represent clusters. ***A.*** Responses from C1, containing 53 autistic and 18 typically-developing participants. ***B.*** Responses from C2, containing 24 autistic and 17 typically-developing participants. ***C*.** Responses from C3, containing 32 autistic and 31 typically-developing participants. ***D.*** Responses from C4, containing 23 autistic and 15 typically-developing participants.


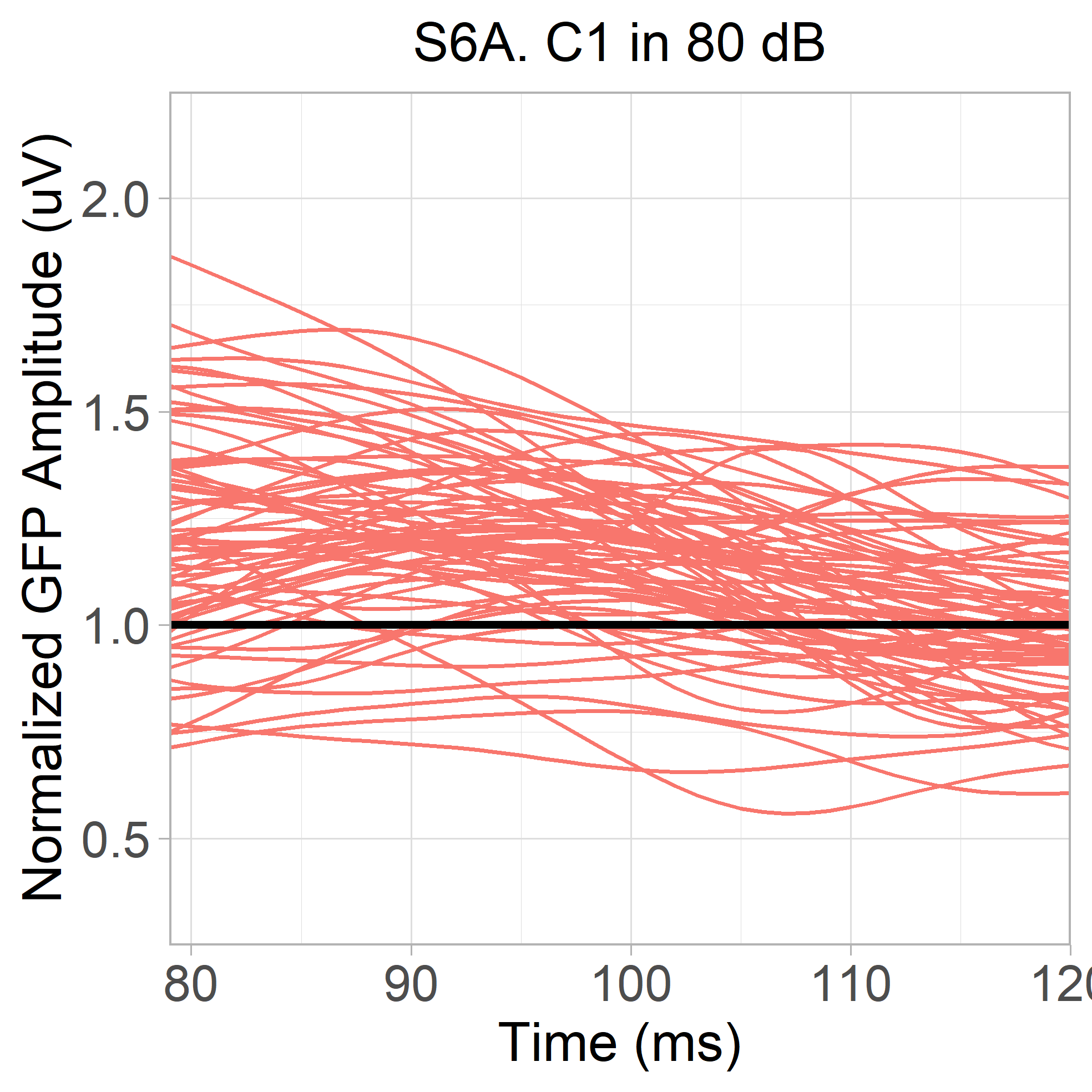

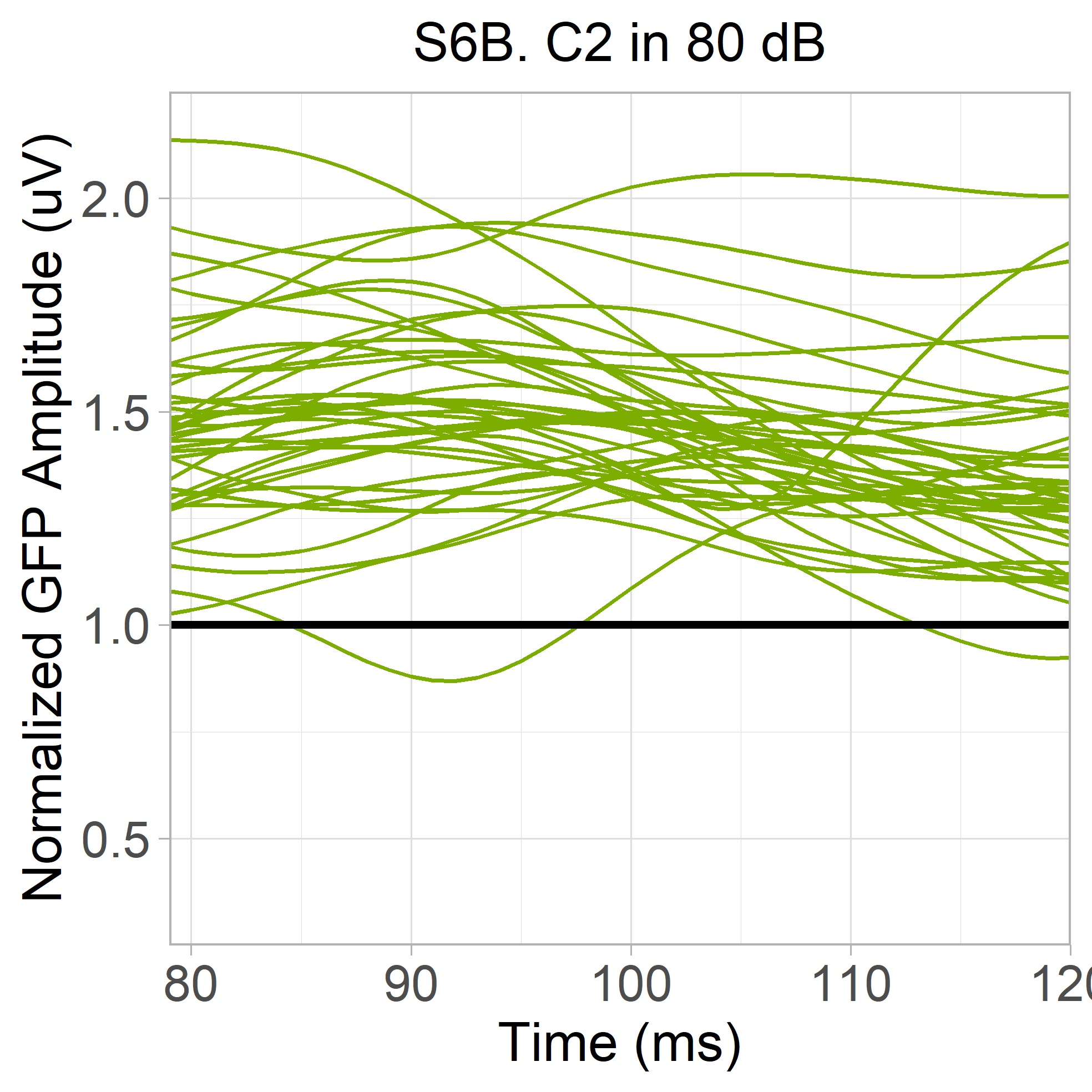


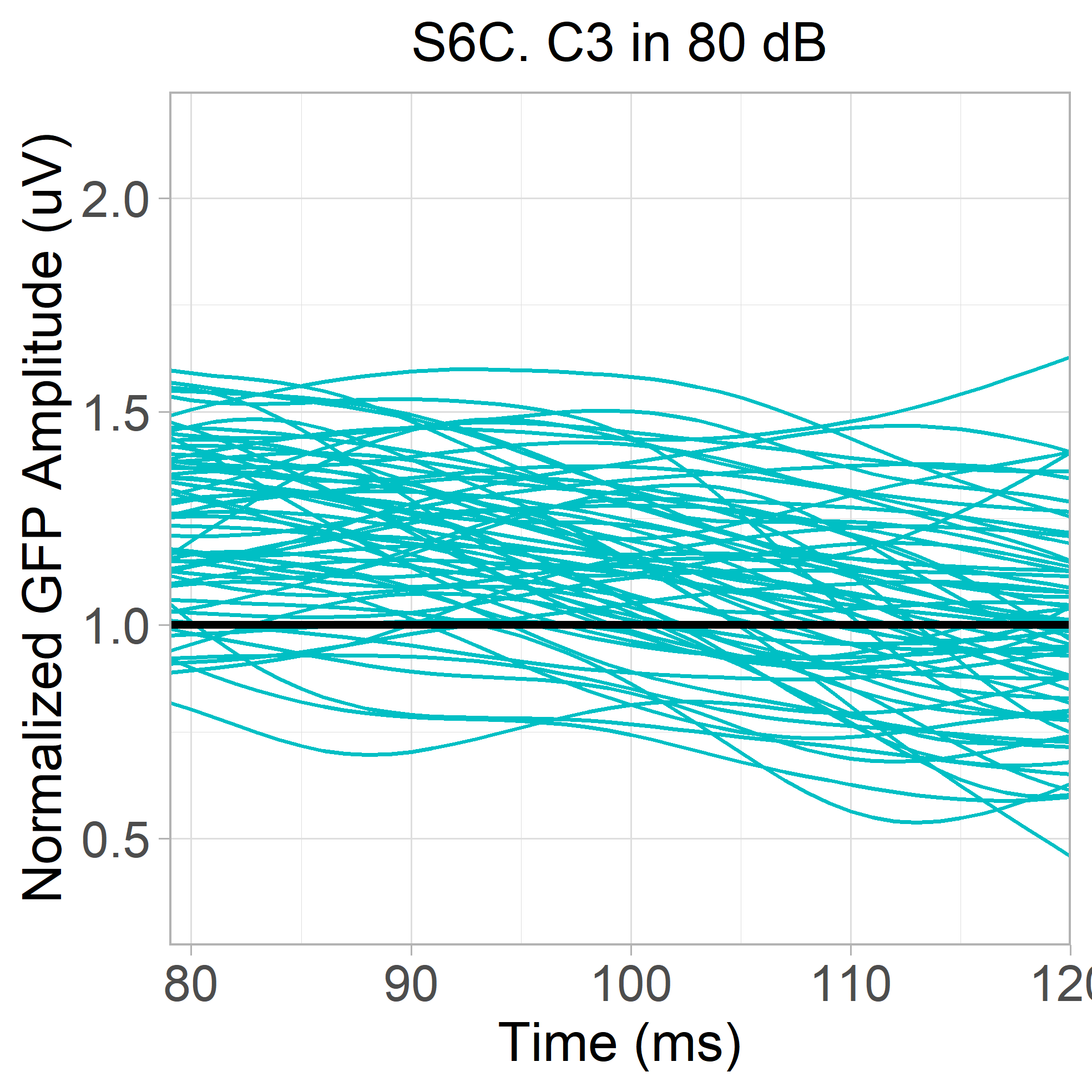

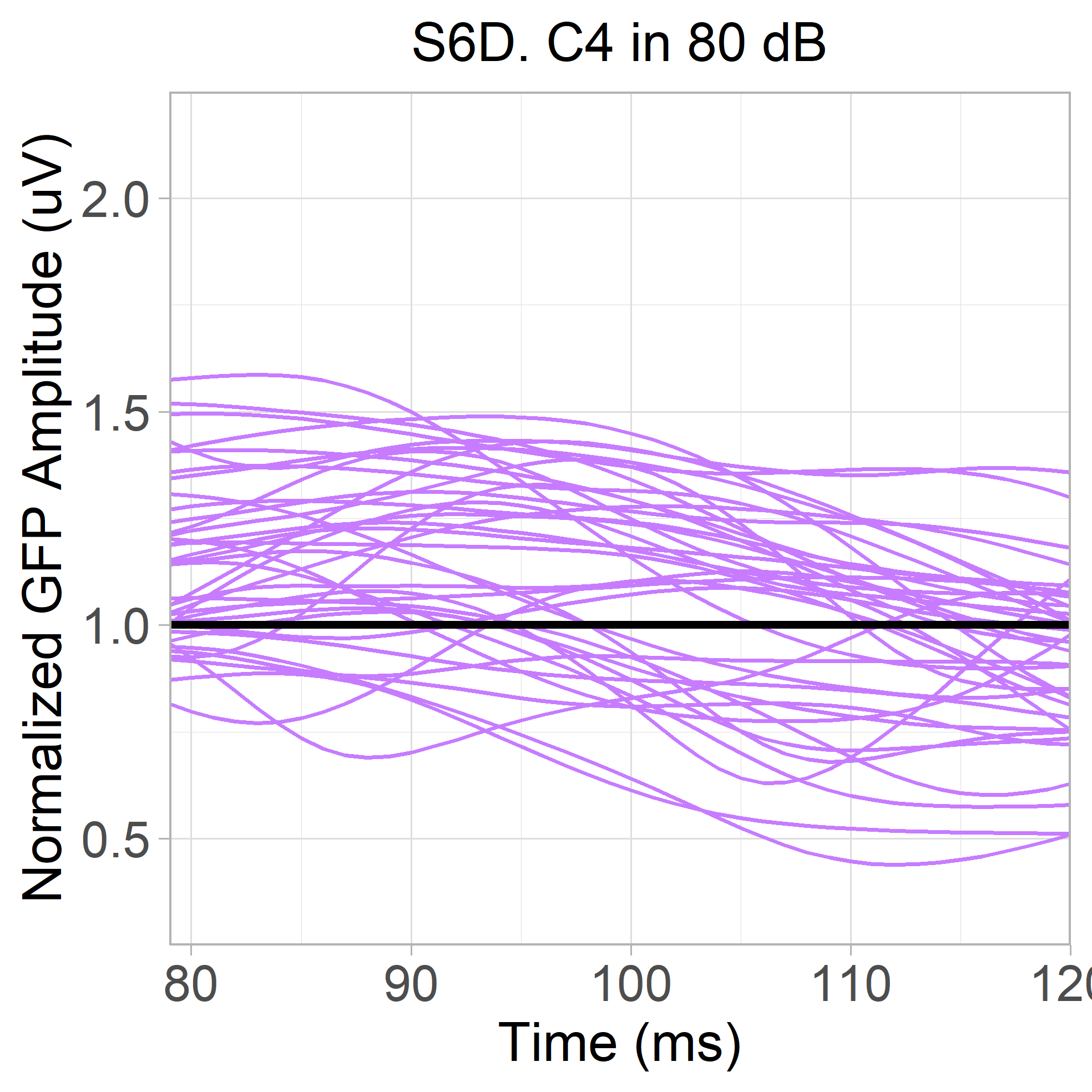


*Figure S6*. Spaghetti plot of normalized GFP waveforms from each participant in the 80 dB condition (79 – 120ms). Colours represent clusters. ***A.*** Responses from C1, containing 53 autistic and 18 typically-developing participants. ***B.*** Responses from C2, containing 24 autistic and 17 typically-developing participants. ***C*.** Responses from C3, containing 32 autistic and 31 typically-developing participants. ***D.*** Responses from C4, containing 23 autistic and 15 typically-developing participants.

1. It should be noted that simply splitting these participants into a separate cluster was not appropriate, given that they occupied a lower rung of the cluster hierarchy. Separating these participants into their own cluster would have increased the number of clusters to at least fourteen, and perhaps as many as seventeen, depending on the choice of boundary. [↑](#footnote-ref-1)
